# *Circ-Ntrk2* acts as a *miR-296-5p* sponge to activate the TGF-β1/p38 MAPK pathway and promote pulmonary hypertension and vascular remodelling

**DOI:** 10.1186/s12931-023-02385-7

**Published:** 2023-03-13

**Authors:** Lihuang Su, Xiuchun Li, Xulong Mao, Tingting Xu, Yiying Zhang, Shini Li, Xiayan Zhu, Liangxing Wang, Dan Yao, Jian Wang, Xiaoying Huang

**Affiliations:** 1 Division of Pulmonary Medicine, Wenzhou Key Laboratory of Interdiscipline and Translational Medicine, The First Affiliated Hospital of Wenzhou Medical University, Wenzhou, 325000 Zhejiang China; 2 State Key Laboratory of Respiratory Disease, National Clinical Research Center for Respiratory Disease, Guangdong Key Laboratory of Vascular Disease, Guangzhou Institute of Respiratory Health, The First Affiliated Hospital of Guangzhou Medical University, Guangzhou, 510000 Guangdong China; 3 Section of Physiology, Division of Pulmonary, Critical Care and Sleep Medicine, University of California, La Jolla, San Diego, CA USA

**Keywords:** *Circ-Ntrk2*, Pulmonary arterial hypertension, Vascular remodelling, *miR-296-5p*, Proliferation, Right ventricular hypertrophy

## Abstract

**Background:**

Circular RNAs (circRNAs), a novel class of non-coding RNAs, play an important regulatory role in pulmonary arterial hypertension (PAH); however, the specific mechanism is rarely studied. In this study, we aimed to discover functional circRNAs and investigate their effects and mechanisms in hypoxia-induced pulmonary vascular remodelling, a core pathological change in PAH.

**Methods:**

RNA sequencing was used to illustrate the expression profile of circRNAs in hypoxic PAH. Bioinformatics, Sanger sequencing, and quantitative real-time PCR were used to identify the ring-forming characteristics of RNA and analyse its expression. Then, we established a hypoxia-induced PAH mouse model to evaluate circRNA function in PAH by echocardiography and hemodynamic measurements. Moreover, microRNA target gene database screening, fluorescence in situ hybridisation, luciferase reporter gene detection, and western blotting were used to explore the mechanism of circRNAs.

**Results:**

RNA sequencing identified 432 differentially expressed circRNAs in mouse hypoxic lung tissues. Our results indicated that *circ-Ntrk2* is a stable cytoplasmic circRNA derived from *Ntrk2* mRNA and frequently upregulated in hypoxic lung tissue. We further found that *circ-Ntrk2* sponges *miR-296-5p* and *miR-296-5p* can bind to the 3′-untranslated region of transforming growth factor-β1 (TGF-β1) mRNA, thereby attenuating TGF-β1 translation. Through gene knockout or exogenous expression, we demonstrated that *circ-Ntrk2* could promote PAH and vascular remodelling. Moreover, we verified that *miR-296-5p* overexpression alleviated pulmonary vascular remodelling and improved PAH through the TGF-β1/p38 MAPK pathway.

**Conclusions:**

We identified a new circRNA (*circ-Ntrk2*) and explored its function and mechanism in PAH, thereby establishing potential targets for the diagnosis and treatment of PAH. Furthermore, our study contributes to the understanding of circRNA in relation to PAH.

**Supplementary Information:**

The online version contains supplementary material available at 10.1186/s12931-023-02385-7.

## Background

Pulmonary arterial hypertension (PAH) is a progressive and fatal cardiopulmonary disease characterised by irreversible pulmonary vascular remodelling, wherein right heart hypertrophy and heart failure are the leading causes of death [1]. Pulmonary artery smooth muscle cell (PASMC) over-proliferation is involved in PAH development, thus playing a crucial role in pulmonary vasculature reconstruction owing to its excellent ability to regulate contraction and synthesis dynamically [2]. Regrettably, the current PAH therapies mainly aim to enhance the NO and prostacyclin pathways and inhibit the endothelin pathway. However, only symptoms of the disease can be alleviated through this approach, failing to effectively reverse the hallmark pathological changes in pulmonary vascular remodelling. Consequently, the five-year mortality rate of PAH remains high [3, 4]. Therefore, finding novel targets to inhibit PASMC over-proliferation is crucial.

In recent years, non-coding RNAs (ncRNAs) have been widely reported to play an important role in the development of PAH and other human diseases [5]. Circular RNAs (circRNAs), as a class of highly conserved regulatory ncRNAs, are derived from exons, introns, or intergenic regions through back-splicing [6, 7]. Numerous studies have reported that circRNA can sponge microRNA (miRNA) to alter physiological and pathological states in organisms [8–10]. Due to their unique covalent closed-loop structure, circRNAs exhibit superior stability compared with linear RNA [11–13]. Combined with the strong species preservation, tissue specificity, and expression abundance in mammals, circRNAs hold promise as ideal biomarkers for disease diagnosis and intervention [12–14]. CircRNA function and mechanism in PAH have attracted increasing attention in recent years [15].

However, studies on the function and mechanism of circRNA in PAH are still rare [16, 17]. Therefore, constructing the circRNA expression profile in this disease and identifying new functional circRNAs are of great significance for understanding their role in PAH and developing new therapeutic targets.

In this study, we compared the circRNA expression profile in the lung tissues of normoxic and hypoxic mice. We further investigated the function and mechanism of the newly identified circRNA in PAH at both the in vitro and in vivo levels, suggesting its potential application as a novel therapeutic target for PAH.

## Methods

### Reagents

The primary antibodies used in the present study are shown in Additional file 2: Table S1.

### Cell culture and treatment

Male C57BL/6 mice (6–8 weeks old, 20–25 g) were used for PASMCs isolation. The mice were anaesthetised via isoflurane inhalation (1–2%, 0.6–1 L/min). The lungs were quickly removed and transferred to a phosphate-buffered saline (PBS) solution containing 1% penicillin and streptomycin (Gibco, USA). Under an anatomical microscope (NIKON, Japan), the lung tissue around the pulmonary artery was cleared first, and then the extravascular fascia was peeled off. Using microforceps, the endothelial cells were scraped from the blood vessel lining. The pulmonary artery was snipped and digested in 0.2% collagenase I (Gibco, USA) at 37 °C for 40 min. The primary cells were cultured in Dulbecco's modified eagle medium (Gibco, USA) supplemented with 20% fetal bovine serum (Gibco, USA) and 1% penicillin and streptomycin at 37 ℃ under 5% CO_2_ for 3–5 days. The third and fourth generations of PASMCs were used in this study. The PASMCs were divided into the following groups: 1. normoxia (NOR), HYP, HYP + si-*circ-Ntrk2* negative control (SI-NC), HYP + si-*circ-Ntrk2* (SI, 50 nM); 2. NOR, HYP, and HYP + *circ-Ntrk2* plasmid treatment NC (T-NC), HYP + *circ-Ntrk2* plasmid treatment (T, 50 nM); 3. NOR, HYP, HYP + *miR-296-5p* inhibitor-NC (inhibitor-NC), HYP + *miR-296-5p* inhibitor (Inhibitor, 100 nM); 4. NOR, HYP, HYP + *miR-296-5p* mimic negative control (Mimic-NC), HYP + Mimic (50 nM); 5. NOR, HYP, HYP + SI-NC + Inhibitor-NC, HYP + SI + Inhibitor-NC, HYP + SI + Inhibitor. The NOR group (21% O_2_, 74% N_2_, and 5% CO_2_) and the HYP group (5% O_2_, 90% N_2_, and 5% CO_2_) were incubated at 37 °C for 48 h.

### Quantitative real-time polymerase chain reaction (qRT-PCR)

Total RNA was extracted by a RNeasy^®^ Mini Kit (QIAGEN, Hilden, Germany). RNase R (Beyotime, Shanghai, China) was used to detect circRNA. Genomic DNA (gDNA) was isolated using a Genomic DNA Extraction Kit (Omega Bio-Tek, Norcross, GA, USA). Quantification of RNA and gDNA was performed using the TOROIVD^®^ qRT Master Mix and SYBR Green qPCR Master Mix (TOROIVD, Shanghai, China). miRNA was isolated using the miRNA Extraction Kit (Sangon Biotech, Shanghai, China), and a Bulge-Loop miRNA qRT-PCR Starter Kit (RiboBio, Guangzhou, China) was used to perform real-time PCR. The circRNA and mRNA levels were normalised to those of *β-actin*, and miRNA was normalised to that of *U6* and determined using the 2^−ΔΔCt^ method. The primer sequences used in this study are shown in Additional file 3: Table S2.

### Immunofluorescence

Lung tissues were fixed using a tissue fixator overnight, permeated with 0.1% Triton X-100, and then blocked with 5% bovine serum albumin. Then, sections were incubated with anti-alpha-smooth muscle actin antibodies (1:100) overnight at 4 °C. After rinsing the sections with PBS three times, we incubated the sections with fluorescence-conjugated secondary antibodies. The nuclei were counterstained with 4,6-diamidino-2-phenylindole (DAPI). The fluorescence microscope (Leica DMi8, Wetzlar, Germany) was used to capture fluorescent images.

### Western blot assay

Lung tissues were homogenised with a Protein Extraction Reagent (BOSTER, Wuhan, China) using an automatic homogeniser (FastPrep-24 5G, MP Biomedicals, Santa Ana, CA, USA). Supernatants were collected after the homogenates were centrifuged (12,000 rpm, 4 °C) for 30 min. PASMC proteins were extracted with Protein Extraction Reagent (BOSTER). Western blotting was performed as previously described [18] using antibodies specific for PCNA (1:1000), cyclin D1 (1:1000), transforming growth factor-β1 (TGF-β1) (1:1000), p38 MAPK (1:1000), and β-tubulin (1:1000).

### Proliferation assays

Cells were incubated in 96-well plates for 48 h with a density of 1 × 10^4^ cells per well. Cell proliferation was analysed with 5-ethynyl-20-deoxyuridine (EdU, Abcam, Waltham, MA, USA) and cell counting kit-8 (CCK8, Dojindo, Japan) assay kits.

### Sequencing analysis

Total RNA was isolated, as mentioned above. After the completion of product inspection, the NEBNext^®^ Multiplex Small RNA Library Prep Set for Illumina (NEB, Ipswich, MA, USA) was used for sRNA library construction. Then, the sRNA libraries were sequenced by the HiSeq 2500 platform (Illumina, San Diego, CA, USA).

### RNA target prediction

Putative circRNA and miRNA targets were identified by analysis tools, including circBase (http://www.circbase.org/), miRanda (http://www.microrna.org/microrna/home.do), TargetScan (http://www.targetscan.org/), miRWalk (http://www.ma.uni-heidelberg.de/apps/zmf/mirwalk/), miRDB (http://mirdb.org/miRDB/), and miRTarBase (http://mirtarbase.mbc.nctu.edu.tw/index.html).

### Dual luciferase reporter assay

Dual luciferase reporter analysis of potential binding sites between *circ-Ntrk2* and *miR-296-5p* was performed using the miRanda web tool, whereas targeted binding between *miR-296-5p* and TGF-β1 was analysed using TargetScan, miRDB, miRTarBase, and miRWalk web tools. *Circ-Ntrk2* wild-type (*circ-Ntrk2-WT*) and *circ-Ntrk2* mutant (*circ-Ntrk2-MUT*) carrying the mutated binding sites for *miR-296-5p*, as well as TGF-β1 wild-type (TGF-β1-WT) and TGF-β1 mutant carrying the mutated binding sites for *miR-296-5p* (TGF-β1-MUT) were cloned into the pmiR-RB-ReportTM vector (RiboBio); the primers are shown in Additional file 3: Table S2. PASMCs were transfected with 100 ng pmirGLO vector, 50 nM miR-296-5p mimics, and their negative control (miR-NC) using Attractene Transfection Reagent (Qiagen, Germantown, MD, USA). After 48 h, the Dual Luciferase^®^ Reporter Assay System (E1910, Promega, Madison, WI, USA) was used to measure firefly and Renilla luciferase activities.

### Oligonucleotide design and transfection

For *circ-Ntrk2* and *miR-296-5p* knockdown or overexpression, small interfering RNA (siRNA, si-*circ-Ntrk2*), *circ-Ntrk2* plasmid, *miR-296-5p* inhibitor, and mimic were obtained from RiboBio. The siRNA, mimic, and inhibitor were transfected using the Ribo FECT™ CP transfection kit (RiboBio). Attractene Transfection Reagent (Qiagen, USA) was used for plasmid transfection. The sequences used are shown in Additional file 3: Table S2.

### Animal models

Male C57BL/6 mice (6–8 weeks, 20–25 g) were obtained from Vital River Laboratory Animal Technology (Beijing, China). To verify the function of *circ-Ntrk2*, we randomly divided the mice into four groups: NOR, HYP, HYP + SI-NC, and HYP + SI (20 nmol, i.p.). To identify the function of *miR-296-5p*, we randomly divided the mice into four groups: NOR, HYP, HYP + *miR-296-5p* agomir-NC (Agomir-NC), and HYP + Agomir (20 nmol, i.p.). To investigate whether *circ-Ntrk2* aggravated HYP-induced PAH by inhibiting the negative regulatory effects of *miR-296-5p* on TGF-β1, we randomly divided the mice were into five groups: NOR, HYP, HYP + SI-NC + *miR-296-5p* antagomir NC (ANT-NC), HYP + SI + ANT-NC, and HYP + SI + ANT (50 nmol, i.p.). The HYP group mice were raised by supplying 10% O_2_ for 21 days, while the NOR group was housed in ambient air.

### Echocardiography and measurement of hemodynamic

The mice were anaesthetised via isoflurane inhalation (1–2%, 0.6–1 L/min) and were allowed to moult. High-Resolution Small Animal Ultrasound Imaging System (Vevo 3100, Fujifilm Visual Sonics, Canada) was used to measure the pulmonary artery velocity time integral (PAVTI) and pulmonary artery acceleration time (PAAT). PowerLab 8/35 Multichannel Biological Signal Recording System (AD Instruments, Bella Vista, AUS) was used to record right ventricular systolic pressure (RVSP). After the mice were sacrificed, the right ventricle (RV) was dissected, and the weights of the RV, septum (S), and left ventricle (LV) were recorded. The ratio of RV to (LV + S) weight was calculated to assess the degree of RV hypertrophy.

### Measurement of pulmonary vascular remodelling and collagen deposition

Lung tissue sections were stained with haematoxylin and eosin (H&E). Representative pulmonary arteries with diameters ranging from 25 to 100 µm were selected for further analysis. The ratio of pulmonary artery wall thickness (WT) to total thickness (TT, WT/TT) and the ratio of wall area (WA) to total area (TA, WA/TA) were analysed by Image-Pro Plus 6.0 (Media Cybernetics, Bethesda, MD, USA). Sections were stained with Masson’s trichrome. ImageJ 1.8.0.112 software (NIH, Bethesda, MD, USA) was used to calculate the ratio of collagen fibre area to pulmonary artery WA.

### RNA fluorescence in situ hybridisation (FISH)

Digoxin-labelled sense or antisense probes were synthesised for the *circ-Ntrk2* junction sequence (Additional file 3: Table S2). Hybridisation was performed using a FISH kit (RiboBio), and the nuclei were counterstained with DAPI.

### Statistical analysis

GraphPad Prism 7.0 (Graph Pad Software Inc., San Diego, CA, USA) was used for statistical analysis of the data, and the results are presented as mean ± standard deviation (SD). Student’s t-test and one-way analysis of variance were used for two-group comparisons and multiple comparisons, respectively. Statistical significance was set at *P* < 0.05.

## Results

### *Circ-Ntrk2* upregulates in the lung tissues of HYP-induced PAH mice and PASMCs

**Fig. 1:**
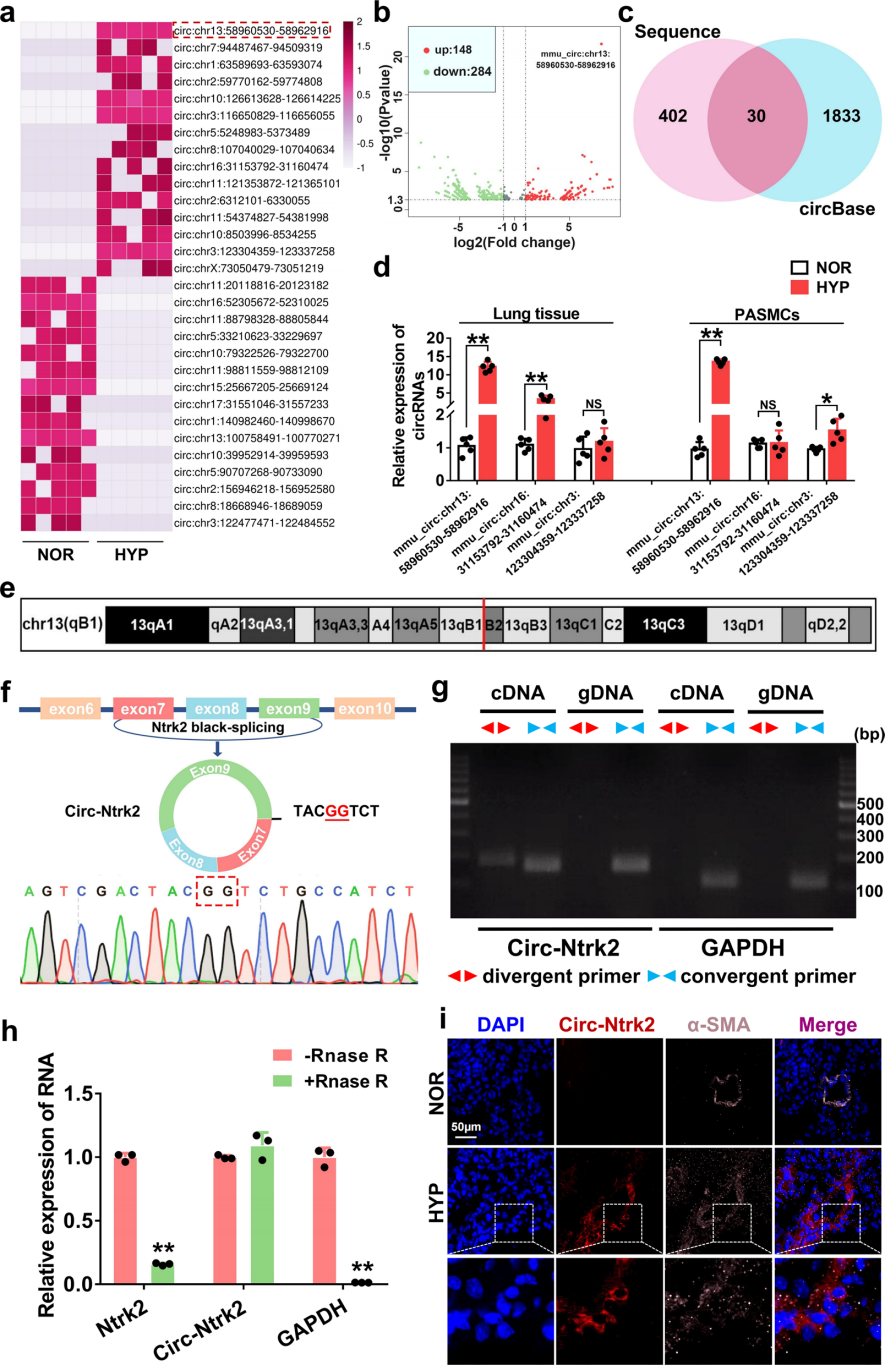
*Circ-Ntrk2* is upregulated in lung tissues of hypoxia-induced PAH mice and PASMCs. **a** Heat map analysis of circular RNA (circRNA) in lung tissues from hypoxia-exposed mice. **b** Volcano map analysis of circRNAs. **c** The differentially expressed circRNAs in the sequencing results were matched with the circBase data. **d** Quantitative polymerase chain reaction (qPCR) analysis of circRNA expression in hypoxic mouse lung tissues and PASMCs (n = 5). **e** Position of *circ-Ntrk2* in its host gene *Ntrk2*. **f** *Circ-Ntrk2* was spliced from the end of the mRNA *Ntrk2* single exon; Sanger sequencing was used to verify the correctness of the cyclization site. **g** PCR assay with divergent and convergent primers showing the amplification of circRNA from cDNA or genomic DNA (gDNA), while *GAPDH* was used as a negative control. The gel or blot images have been cropped and the complete images have been submitted separately (Additional file 4). **h** qRT-PCR analysis of *circ-Ntrk2* and *Ntrk2* mRNA after treatment with RNase R. **i** Fluorescence in situ hybridisation (FISH) of lung tissues of mice exposed to hypoxia. All values are presented as the mean ± SD. *P < 0.05, **P < 0.01

To study the expression profile and functional mechanism of circRNAs in HYP-induced PAH, we constructed a PAH mouse model (Additional file 1: Fig. S1a and b). Further, we screened 432 differentially expressed circRNAs (filtered by log^2^ (fold change >|1|) and *P* < 0.05) in HYP-exposed tissues compared with those in NOR using high-throughput transcriptome sequencing (Fig. 1a and b). Among these circRNAs, 148 were significantly upregulated and 284 were downregulated; mmu_circ: chr13:58960530–58962916 showed the most significant changes. Comparing our results with circBase (http://www.circbase.org/) revealed 30 recorded circRNAs, where mmu_circ: chr13:58960530–58962916 was also found among them*.* Three circRNAs (mmu_circ: chr13:58960530–58962916, mmu_circ: chr16:31,153,792–31,160,474, and mmu_circ: chr3:123304359–123337258) that were stably upregulated under hypoxic conditions were selected for further verification (Fig. 1c). The qPCR results showed that mmu_circ: chr13:58960530–58962916 was most significantly upregulated in hypoxic lung tissues and PASMCs, consistent with the sequencing results (Fig. 1d). Therefore, it was selected as the object for subsequent research. Also, since it is derived from a protein-coding locus *Ntrk2* located on chromosome 13qB1 (Fig. 1e), we named this circRNA “*circ-Ntrk2.*”

### *Circ-Ntrk2* is a stable cytoplasmic circRNA derived from *Ntrk2* mRNA

To further verify the ring structure of *circ-Ntrk2*, we first confirmed its junction site by Sanger sequencing analysis. The genomic structure showed that *circ-Ntrk2* is looped by the seventh and ninth exons of the *Ntrk2* gene (Fig. 1f). *Circ-Ntrk2* was validated via PCR using divergent primers from cDNA rather than gDNA (Fig. 1g). Reverse-transcribed cDNA after RNase R treatment using qPCR showed no significant changes in *circ-Ntrk2* compared with the untreated group, while the *GAPDH* gene and *Ntrk2* mRNA were significantly downregulated, indicating good *circ-Ntrk2* resistance to RNase R, further supporting the possibility of its cyclisation (Fig. 1h). FISH demonstrated that *circ-Ntrk2* was preferentially localised within the PASMC cytoplasm (Fig. 1i). These results indicated that *circ-Ntrk2* is a stable cytoplasmic circRNA derived from *Ntrk2* mRNA.

### *Circ-Ntrk2* knockdown alleviated pulmonary vascular remodelling and improved PAH

**Fig. 2:**
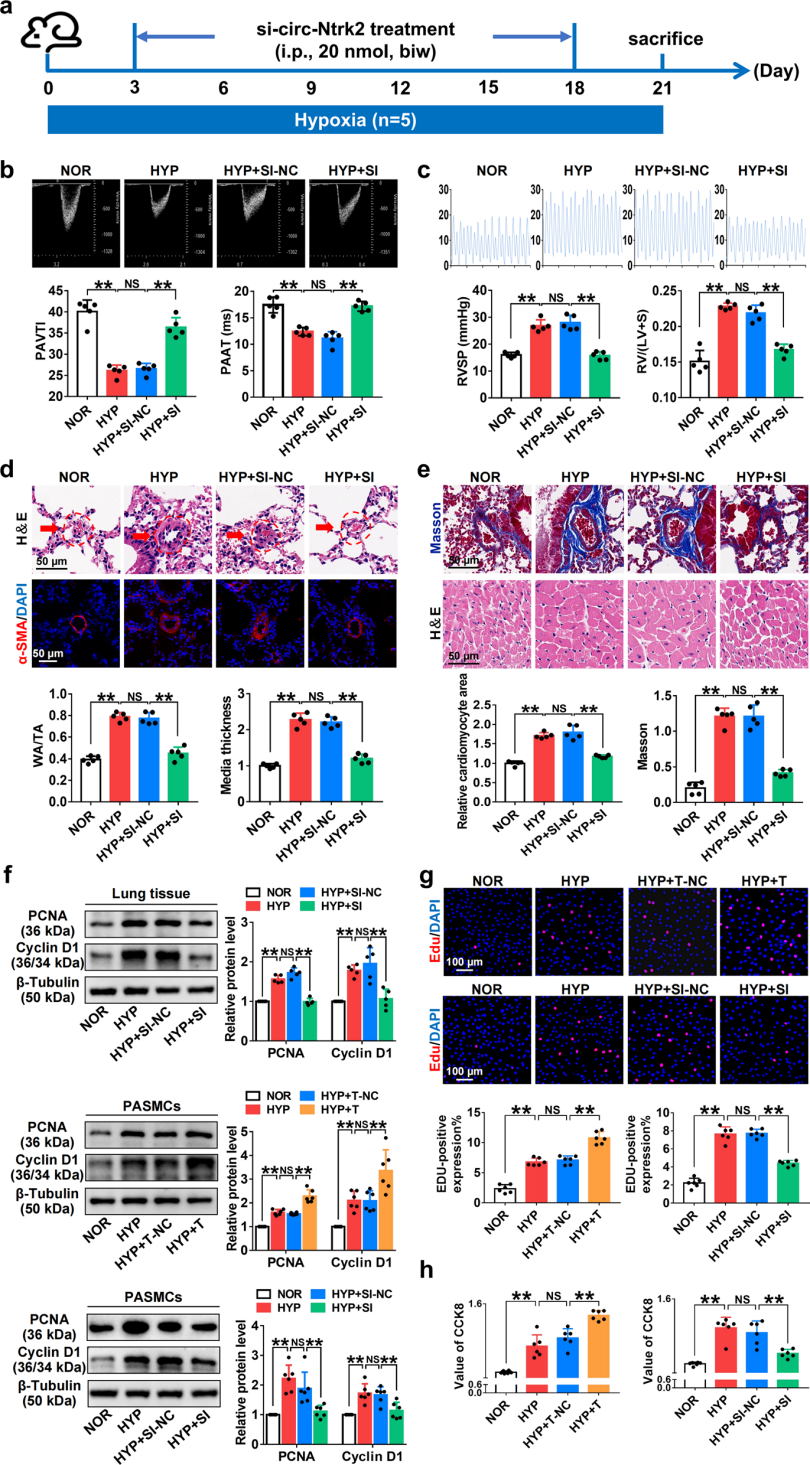
*Circ-Ntrk2* knockdown alleviates pulmonary vascular remodelling and improves PAH. **a** Construction of small interfering RNA (siRNA) based on the target sequence. All treatment groups received injections twice weekly for modelling for 21 days. **b** Echocardiographic measurements and images illustrating pulmonary artery acceleration time (PAAT) and pulmonary arterial velocity time integral (PAVTI) in the NOR, HYP, HYP + SI-NC, and HYP + SI groups are shown (n = 5). **c** Right ventricular systolic pressure (RVSP) and the right ventricle (RV)/left ventricle (LV) + septum (S) weight ratio in the NOR, HYP, HYP + SI-NC, and HYP + SI groups (n = 5). **d** Morphological analysis of the pulmonary artery was performed using H&E staining and immunofluorescence (n = 5). **e** Detection of collagen deposition around blood vessels using Masson staining (up) (n = 5) and H&E staining of myocardial cells in right ventricular wall (bottom) (n = 5). **f** Analysis of PCNA (proliferating cell nuclear antigen) and cyclin D1 expression in lung tissues (n = 5) and PASMCs (n = 6). The gel or blot images have been cropped and the complete images have been submitted separately (Additional file 4). **g** EdU in PASMCs (n = 6). **h** CCK-8 assay in PASMCs (n = 6). All values are presented as the mean ± SD. *P < 0.05, **P < 0.01

To investigate the role of *circ-Ntrk2* in PAH, we knocked down *circ-Ntrk2* using siRNA (Fig. 2a and Additional file 1: Fig. S1c), and the efficiency was determined using qRT-PCR (Additional file 1: Fig. S1d). Echocardiography revealed that PAAT and PAVTI were apparently shorter under HYP, with *circ-Ntrk2* silencing, which was reversed (Fig. 2b). Additionally, *circ-Ntrk2* silencing attenuated the increase in RVSP and RV/(LV + S) induced by HYP (Fig. 2c). H&E staining and immunofluorescence results indicated that silencing *circ-Ntrk2* reversed HYP-induced pulmonary vascular remodelling (Fig. 2d). Masson staining showed that circRNA knockdown improved perivascular collagen deposition in the disease state. In contrast, H&E staining of the right ventricular wall showed that *circ-Ntrk2* knockdown significantly improved HYP-induced right ventricular cardiomyocyte hypertrophy (Fig. 2e). Western blotting verified that knockdown of *circ-Ntrk2* reversed HYP-induced upregulation of proliferation-related proteins such as PCNA and cyclin D1 (Fig. 2f). These results suggested that *circ-Ntrk2* knockdown can inhibit pulmonary vascular remodelling and alleviate PAH. In vitro, CCK8 and EdU results indicated that the proliferation ability of PASMCs increased under HYP, which was inhibited after silencing *circ-Ntrk2* and enhanced after overexpression of *circ-Ntrk2* (Fig. 2g and h). These results indicated that *circ-Ntrk*2 knockdown inhibits PASMCs proliferation in vitro and reverses pulmonary vascular remodelling to alleviate PAH in vivo.

### *Circ-Ntrk2* acts as a sponge for *miR-296-5p*

**Fig. 3:**
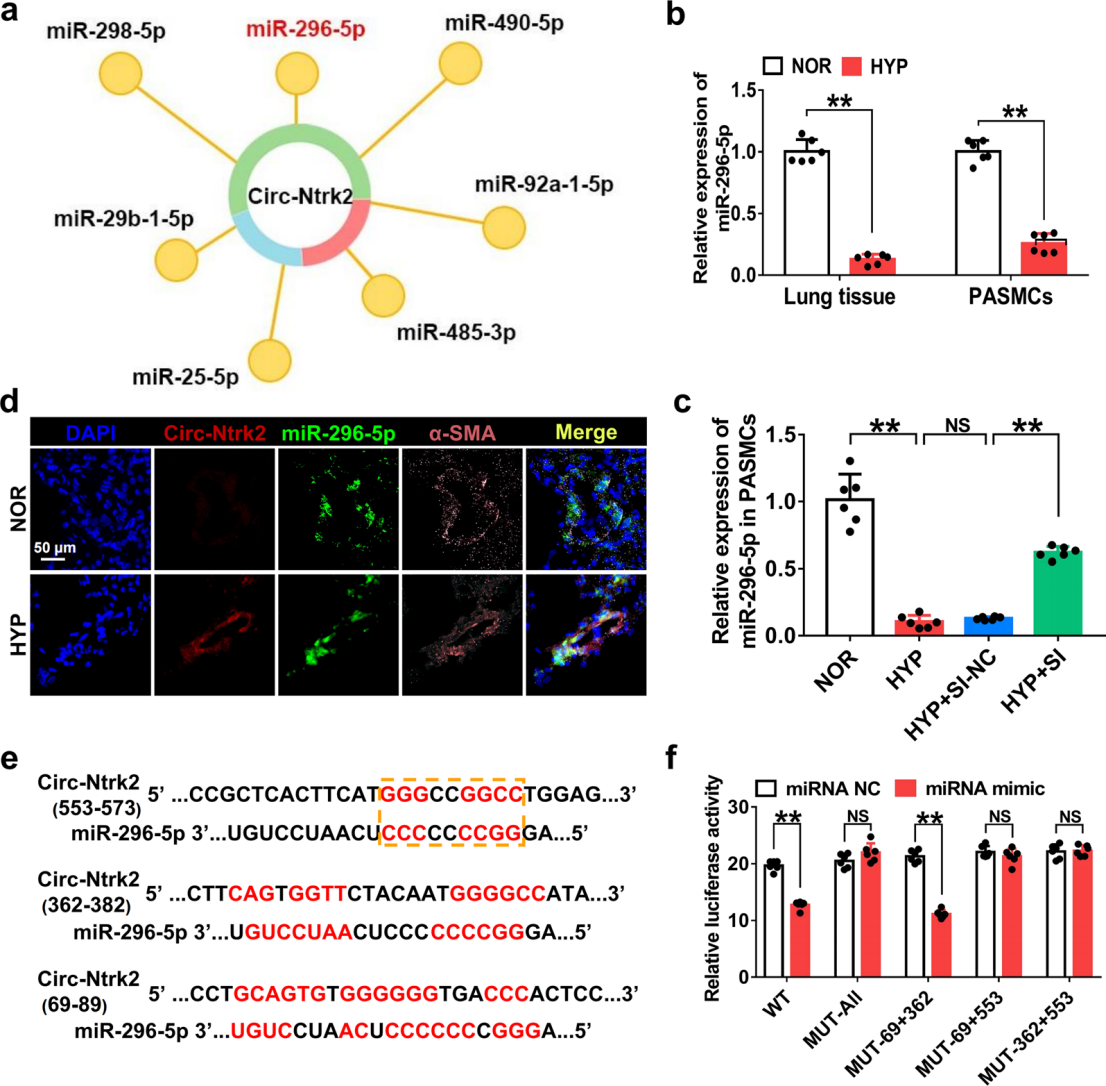
*Circ-Ntrk2* acts as a sponge for *miR-296-5p*. **a** miRanda bioinformatics software predicted miRNAs that could bind to *circ-Ntrk2*. **b** Quantitative polymerase chain reaction (qPCR) analysis of *miR-296-5p* expression in mouse lung tissues and PASMCs (n = 6). **c** PASMCs were transfected with si-*circ-Ntrk2*, and qPCR was used to detect *miR-296-5p* expression (n = 6). **d** Fluorescence in situ hybridization (FISH) was used to observe the colocalization of *circ-Ntrk2* and *miR-296-5p*. **e** TargetScan predicted binding sites and secondary structures of *circ-Ntrk2* and *miR-296-5p*. **f** Dual luciferase vector and mutant plasmids were designed based on binding sites between *circ-Ntrk2* and *miR-296-5p*. Dual luciferase assays were used to validate the interactions between *circ-Ntrk2* and *miR-296-5p* (n = 6). All values are presented as the mean ± SD. *P < 0.05, **P < 0.01

To verify the existence of the circRNA-miRNA axis under the specific pathological conditions of PASMCs, we predicted miRNAs that were probably adsorbed by *circ-Ntrk2* using the bioinformatics software miRanda (http://www.microrna.org/microrna/home.do) (Fig. 3a) and detected their expression by qPCR. Under hypoxic conditions, *miR-296-5p* expression was notably downregulated both in vivo and in vitro (Fig. 3b). PASMCs were transfected with *circ-Ntrk2* siRNA and then exposed to HYP for 48 h. Moreover, under the same conditions, the downregulation of miRNA expression was reversed after the knockdown of *circ-Ntrk2*, consistent with the competitive endogenous RNA mechanism (Fig. 3c); we therefore used *miR-296-5p* in subsequent analyses. To verify whether *circ-Ntrk2* binds to *miR-296-5p*, we performed FISH and showed that *circ-Ntrk2* could co-localise with *miR-296-5p* in the cytoplasm of PASMCs (Fig. 3d). We then predicted that there were three possible target sequence regions between *circ-Ntrk2* and *miR-296-5p* using TargetScan (http://www.targetscan.org/) (Fig. 3e). Subsequently, we constructed dual luciferase vector plasmids and found that the ratio of firefly to Renilla luciferase for wild-type and target sites (position 553–573) without mutant *circ-Ntrk2* was visibly decreased compared with those of the NC, whereas those of the target site with mutant *circ-Ntrk2* did not change (Fig. 3f). These results indicated that *circ-Ntrk2* could act as a sponge for *miR-296-5p*.

### *MiR-296-5p* overexpression alleviated pulmonary vascular remodelling and improved PAH

**Fig. 4:**
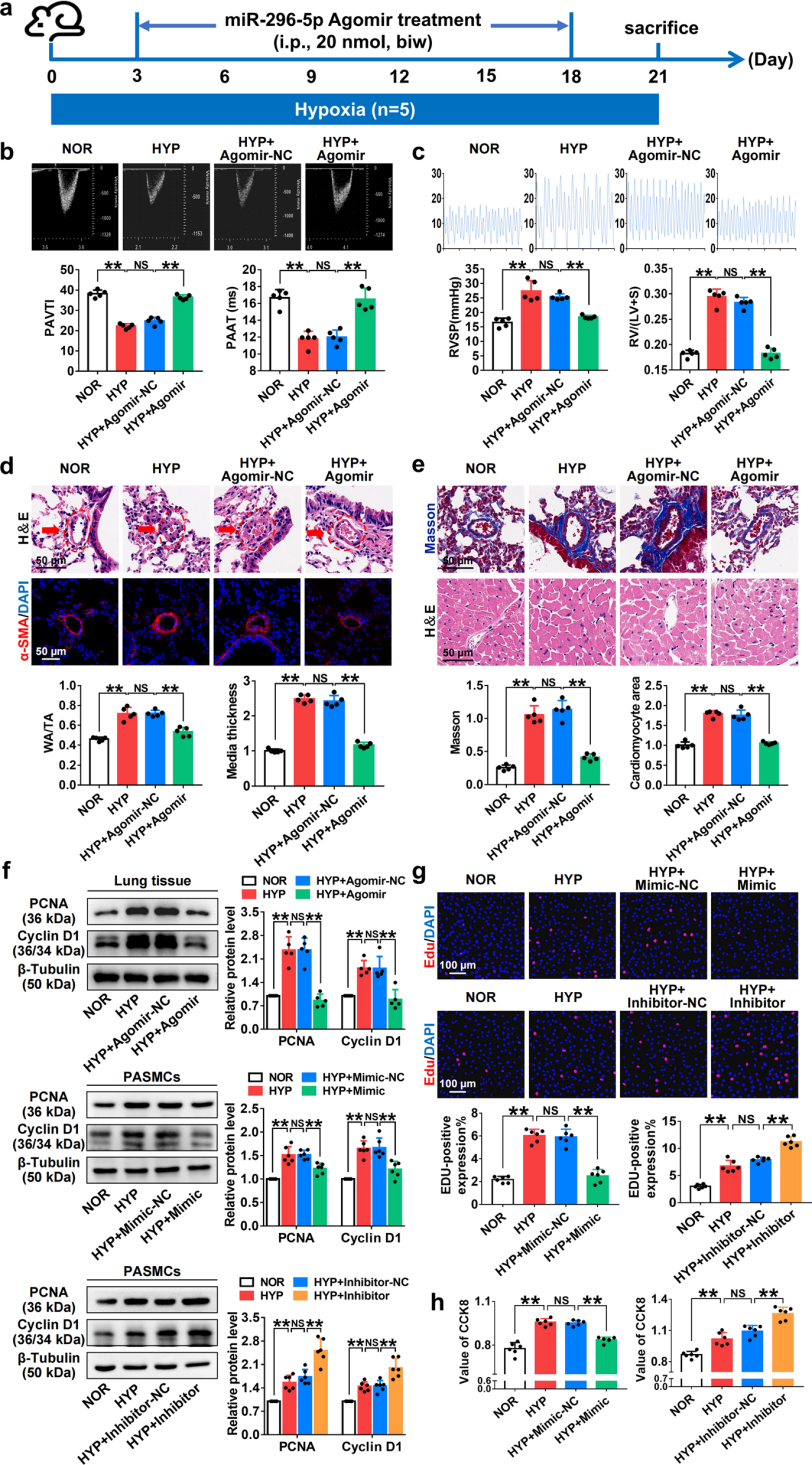
*MiR-296-5p* overexpression alleviates pulmonary vascular remodelling and improves PAH. **a** Agomir constructed for *miR-296-5p* was used to upregulate *miR-296-5p*. All treatment groups received injections twice weekly for modelling for 21 days. **b** Echocardiographic measurements and images illustrating pulmonary artery acceleration time (PAAT) and pulmonary arterial velocity time integral (PAVTI) in the NOR, HYP, HYP + Agomir-NC, and HYP + Agomir groups are shown (n = 5). **c** Right ventricular systolic pressure (RVSP) and the right ventricle (RV)/left ventricle (LV) + septum (S) weight ratio in the NOR, HYP, HYP + Agomir-NC, and HYP + Agomir groups (n = 5). **d** Analysis of pulmonary artery remodelling was performed using H&E staining and immunofluorescence (n = 5). **e** Detection of collagen deposition around blood vessels using Masson staining (up) (n = 5) and H&E staining of myocardial cells in right ventricular wall (bottom) (n = 5). **f** Analysis of PCNA (proliferating cell nuclear antigen) and cyclin D1 expression in the lung tissues (n = 5) and PASMCs (n = 6). The gel or blot images have been cropped and the complete images have been submitted separately (Additional file 4). **g** EdU in PASMCs (n = 6). **h** CCK-8 assay in PASMCs (n = 6). All values are presented as the mean ± SD. *P < 0.05, **P < 0.01

To determine whether *miR-296-5p* plays a role in PAH, we validated its function both in vivo and in vitro. Echocardiography and right cardiac pressure showed that *miR-296-5p* supplementation markedly improved pulmonary arterial pressure under hypoxic condition (Fig. 4a–c, Additional file 1: Fig. S1e). H&E staining and immunofluorescence showed that HYP-induced pulmonary vascular remodelling was alleviated by *miR-296-5p* addition (Fig. 4d). Masson staining showed that *miR-296-5p* could alleviate HYP-induced collagen deposition around the pulmonary artery, and cardiac H&E staining suggested that *miR-296-5p* could modify pathological changes in HYP-induced cardiac hypertrophy (Fig. 4e). Western blotting verified that *miR-296-5p* could reverse HYP-induced upregulation of proliferation-related proteins, such as PCNA and cyclin D1 (Fig. 4f). In vitro, the results of CCK8 and EdU experiments showed that the *miR-296-5p* mimic decreased the EdU incorporation rate and CCK8 value under HYP (Fig. 4g and h). These results suggested that *miR-296-5p* overexpression inhibits HYP-induced proliferation of PASMCs. Taken together, *miR-296-5p* inhibits HYP-induced PASMC proliferation to alleviate pulmonary vascular remodelling and improve PAH.

### *Circ-Ntrk2* sponges *miR-296-5p* to promote pulmonary vascular remodelling and PAH

**Fig. 5:**
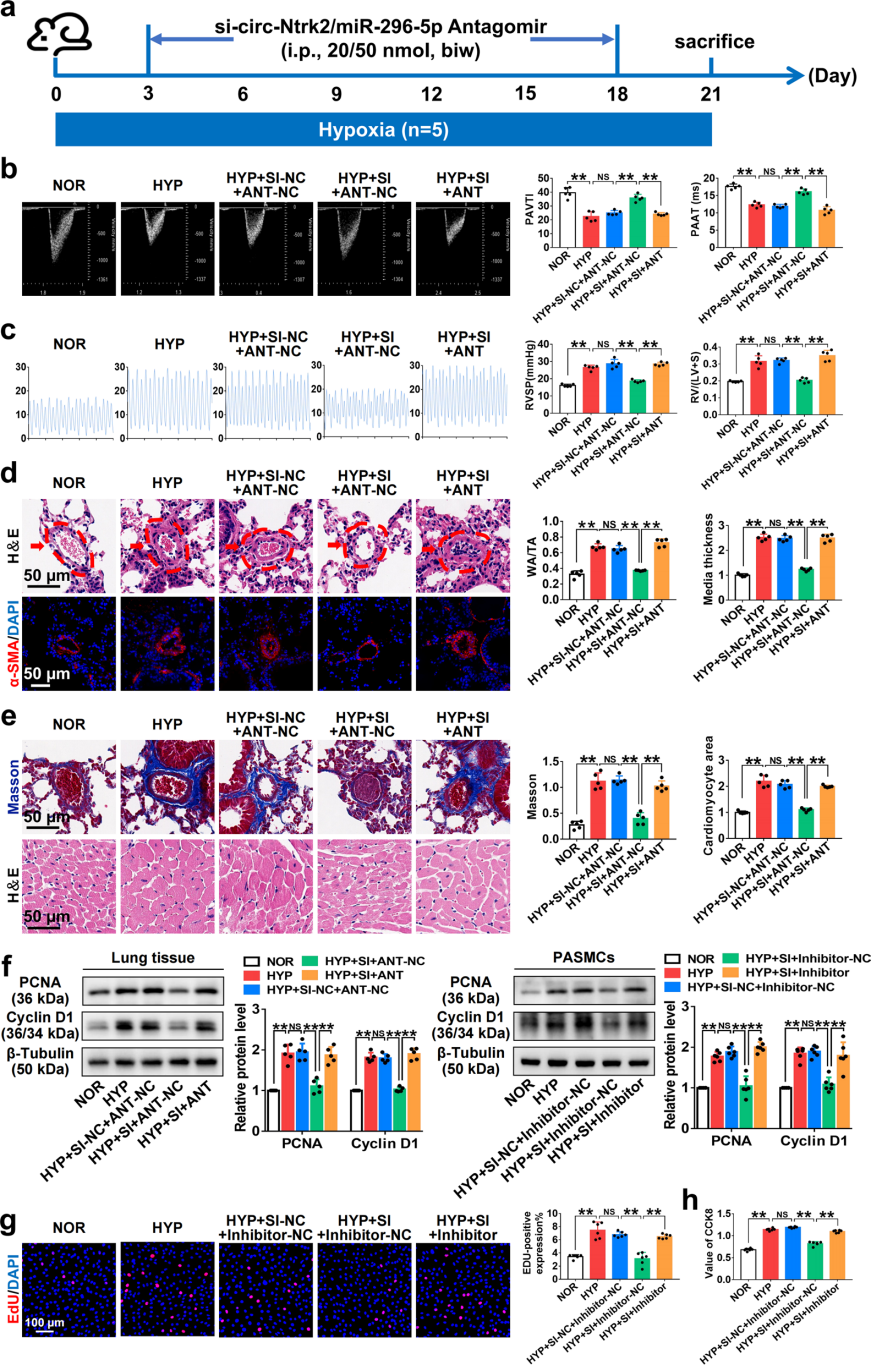
*Circ-Ntrk2* sponges *miR-296-5p* to promote pulmonary vascular remodelling and PAH. **a** All treatment groups received si-*circ-Ntrk2* or *miR-296-5p* antagomir injections twice weekly for modelling for 21 days. **b** Echocardiographic measurements and images illustrating pulmonary artery acceleration time (PAAT) and pulmonary arterial velocity time integral (PAVTI) in the NOR, HYP, HYP + SI-NC + ANT-NC, HYP + SI + ANT-NC, and HYP + SI + ANT groups are shown (n = 5). **c** Right ventricular systolic pressure (RVSP) and the right ventricle (RV)/left ventricle (LV) + septum (S) weight ratio in the NOR, HYP, HYP + SI-NC + ANT-NC, HYP + SI + ANT-NC, and HYP + SI + ANT groups (n = 5). **d** Analysis of pulmonary artery remodelling was performed using H&E and immunofluorescence. **e** Detection of collagen deposition around blood vessels using Masson staining (up) (n = 5) and H&E staining of myocardial cells in right ventricular wall (bottom) (n = 5). **f** Analysis of PCNA (proliferating cell nuclear antigen) and cyclin D1 expression in the lung tissues (n = 5) and PASMCs (n = 6). The gel or blot images have been cropped and the complete images have been submitted separately (Additional file 4). **g** EdU in PASMCs (n = 6). **h** CCK-8 assay in PASMCs (n = 6). All values are presented as the mean ± SD. *P < 0.05, **P < 0.01

To further confirm whether *circ-Ntrk2* affected PAH through *miR-296-5p*, we treated cells with circRNA and miRNA in vitro and in vivo and detected the phenotypic and pathological changes. Echocardiography and right cardiac pressure showed that the moderating effect of *circ-Ntrk2* knockdown on pulmonary artery pressure under HYP was reversed by the *miR-296-5p* antagomir (Fig. 5a–c, Additional file 1: Fig. S1f). Similarly, H&E staining and immunofluorescence showed that the alleviating effect of *circ-Ntrk2* knockdown on HYP-induced pulmonary vascular remodelling was also reversed by the *miR-296-5p* antagomir (Fig. 5d). Moreover, cardiac H&E staining suggested that *circ-Ntrk2* knockdown could correct the pathological changes in HYP-induced myocardial hypertrophy and could be inhibited after exogenous supplementation with *miR-296-5p* antagomir. Masson’s trichrome staining showed a similar trend in collagen deposition (Fig. 5e). Consistently, *miR-296-5p* reversed the regulation of *circ-Ntrk2* on the expression of PCNA and cyclin D1 under HYP (Fig. 5f). In in vitro recovery experiments, including EdU and CCK8, results showed that *circ-Ntrk2* siRNA inhibited the HYP-induced proliferation of PASMCs, whereas the *miR-296-5p* inhibitor reversed this effect (Fig. 5g and h). *Circ-Ntrk2* could promote PASMC proliferation and pulmonary vascular remodelling to regulate PAH progression by adsorbing *miR-296-5p*.

### ***Circ-Ntrk2*** sponges ***miR-296-5p*** to activate the TGF-β1/p38 MAPK pathway and promotes pulmonary vascular remodelling and PAH

**Fig. 6:**
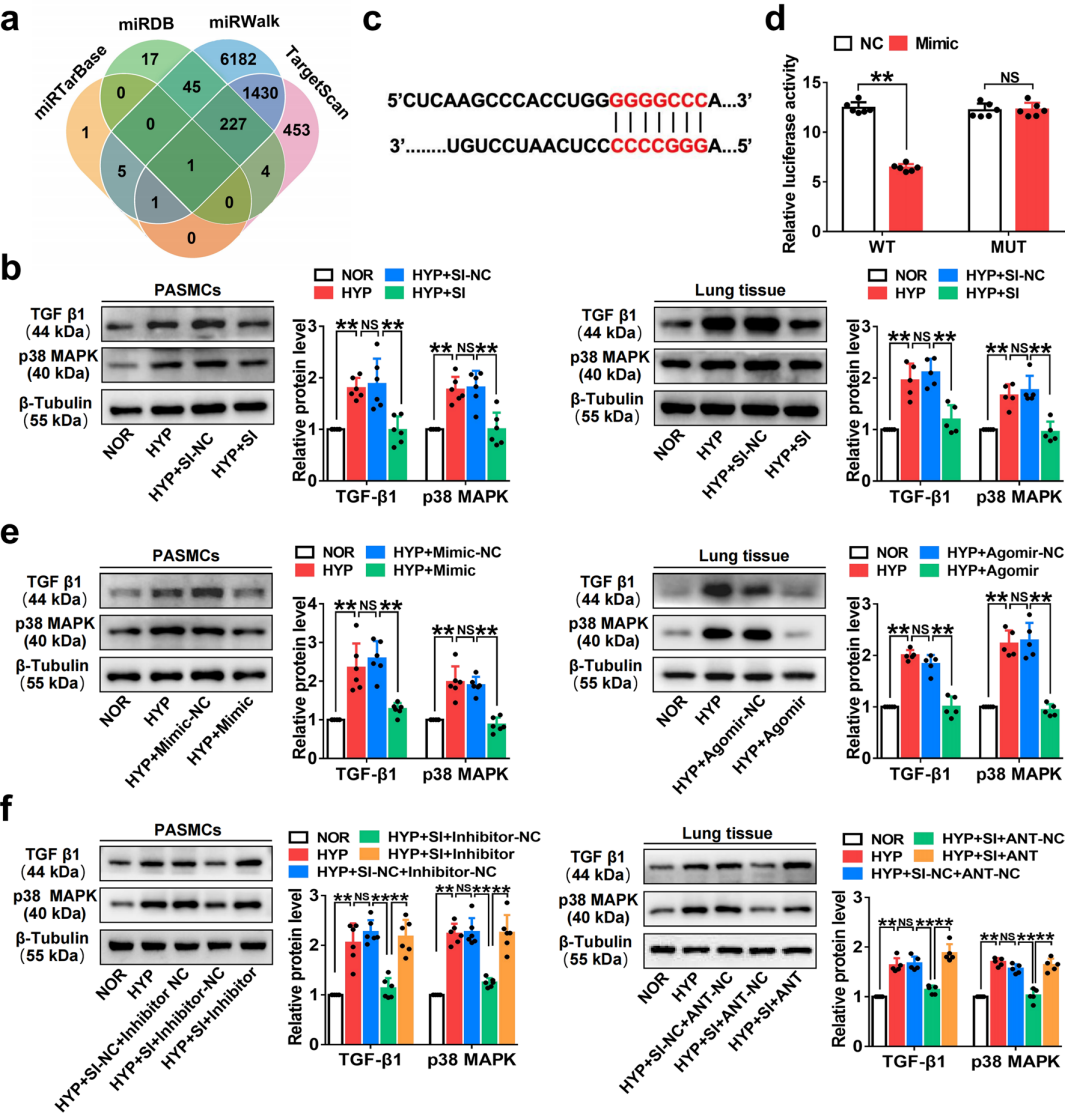
*Circ-Ntrk2* might promote PAH through the *miR-296-5p*/TGF-β1 axis. **a** TargetScan, miRDB, miRTarBase, and miRWalk bioinformatics software predicted *miR-296-5p* target proteins. **b** Western blotting was used to verify the expression of TGF-β1 and p38 MAPK in lung tissues (n = 5) and PASMCs (n = 6) from the NOR, HYP, HYP + SI-NC, and HYP + SI groups. **c** TargetScan predicted binding sites and secondary structures of *miR-296-5p* and TGF-β1. **d** Dual luciferase vector and mutant plasmids were designed based on binding sites between *miR-296-5p* and TGF-β1. Dual luciferase assays were used to validate the interactions between *miR-296-5p* and TGF-β1 (n = 6). **e** Analysis expression of TGF-β1 and p38 MAPK in lung tissues (n = 5) and PASMCs (n = 6) after intervention of *miR-296-5p*. **f** Analysis expression of TGF-β1 and p38 MAPK in lung tissues (n = 5) and PASMCs (n = 6) after co-intervention of *miR-296-5p* and *circ-Ntrk2*. The gel or blot images have been cropped and the complete images have been submitted separately (Additional file 4). All values are presented as the mean ± SD. *P < 0.05, **P < 0.01

**Fig. 7:**
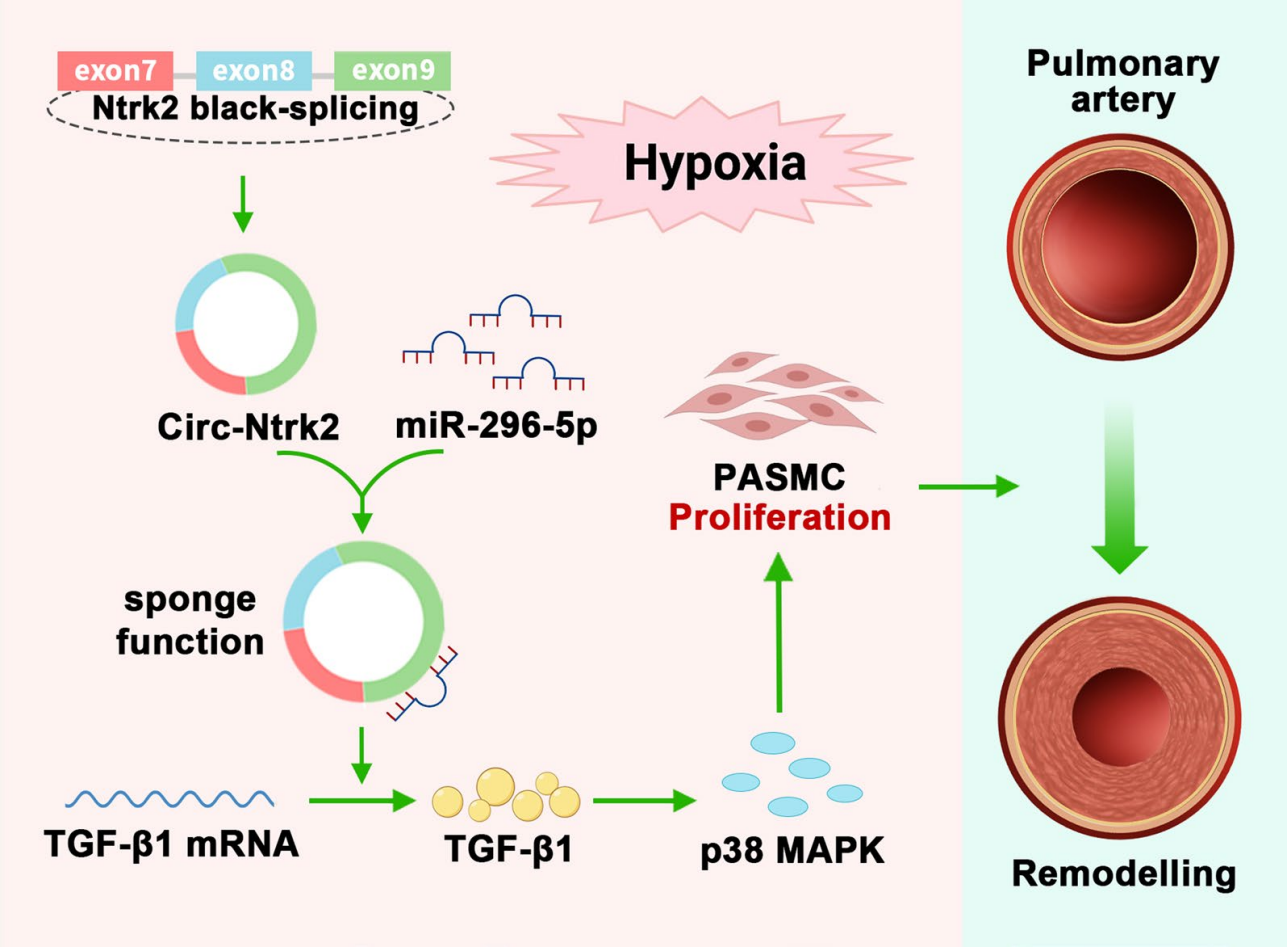
Potential molecular mechanisms of *circ-Ntrk2* in PAH. *Circ-Ntrk2* functions as an accelerator of PAH by sponging *miR-296-5p* to activate the TGF-β1/p38 MAPK pathway, and consequently promotes PASMC proliferation and pulmonary artery remodelling

Bioinformatics software was used to predict the possible target proteins of *miR-296-5p* (Fig. 6a). Our study revealed that TGF-β1 expression was significantly increased under HYP, and its upregulation was inhibited in *circ-Ntrk2* knockdown cells and that p38 MAPK showed similar changes (Fig. 6b). Therefore, it was selected for follow-up research. Dual luciferase reporter assays showed that the ratio of firefly to Renilla luciferase activity in cells expressing wild-type TGF-β1 was distinctly lower than that in the NC; however, there were no significant changes in cells transfected with a binding site mutant TGF-β1 (Fig. 6c and d). Western blotting showed that transfection with *miR-296-5p* mimics inhibited TGF-β1 and p38 MAPK expression, compared with that in NC. Contrasting results were obtained after transfection with the inhibitor in HYP-induced smooth muscle cells (Fig. 6e). In further experiments, we found that *circ-Ntrk2* knockdown could inhibit TGF-β1 and p38 MAPK expression under HYP; however, this effect was reversed after *miR-296-5p* downregulation (Fig. 6f). Altogether, these results demonstrated that antagonising *circ-Ntrk2* could alleviate PAH by inhibiting PASMC proliferation and reversing pulmonary vascular remodelling through the *miR-296-5p*/TGF-β1/p38 MAPK signal transduction axis (Fig. 7).

## Discussion

In this study, we identified a novel circRNA named *circ-Ntrk2* and evaluated its role and mechanism in PAH pathogenesis. First, *circ-Ntrk2* was upregulated in a mouse model of PAH in the lung tissue. Second, silencing *circ-Ntrk2* inhibited HYP-induced PASMC proliferation and reversed HYP-induced PAH in mice. Third, *circ-Ntrk2* acted as a molecular sponge for *miR-296-5p* to regulate the TGF-β1/p38 MAPK pathway. Overall, our results indicate that *circ-Ntrk2* can sponge *miR-296-5p* to activate the TGF-β1/p38 MAPK signalling axis to promote the progression of PAH and pulmonary vascular remodelling.

PAH is a heterogeneous disease characterised by pulmonary vascular remodelling, which often leads to death from right heart failure [19, 20]. PASMC proliferation and endothelial cell dysfunction are involved in PAH occurrence and development, with the former playing a core pathological role [21–23]. Current research suggests that PAH cannot be completely cured due to the lack of drugs that can effectively reverse pulmonary vascular remodelling [24–26].

NcRNA is a class of transcribed RNA with no or weak protein-coding ability, mainly including circRNAs, long ncRNAs (lncRNAs), and miRNAs [5]. Numerous studies have shown that ncRNAs play a vital role in regulating human diseases, such as PAH [27, 28]. CircRNAs, as a novel class of regulatory ncRNA, showed insensitivity to RNase R and were more stable than lncRNA and miRNA [11–13]. Due to their abundance, species preservation, tissue specificity, stability, and regulatory functional roles, circRNAs have been identified as potential biomarkers and therapeutic targets for various diseases, including PAH [13–15, 29, 30]. However, most circRNAs have not been thoroughly verified experimentally, especially in PAH [31–33]. Previous studies have shown that circRNAs play multiple important roles in cellular physiology by acting as miRNA sponges, templates for protein translation, transcriptional regulators, and RBP-binding molecules [34], with sponge mechanisms being the most widely studied [30]. Jiang et al. [17] identified a novel circRNA named *circ-calm4*, demonstrating its ability to promote PASMC proliferation by sponging *miR-337-3p* and regulating HYP-induced PASMC pyroptosis by sponging *miR-124-3p*. Lu et al. [35] reported that *circ-SMOC1* inhibits metabolic reprogramming in PAH by sponging *miR-329-3p*. Huang et al. [36] found that *hsa_circ_0016070* could accelerate PAH progression by targeting *miR-340-5p*. These results provide strong evidence that circRNAs can regulate PAH by sponging miRNAs and suggest that their role in PAH is worth further research and investment. In this study, we identified a novel functional circRNA localised in the cytosol of PASMCs using high-throughput sequencing, Sanger sequencing, and FISH and named it “*circ-Ntrk2*”. It was confirmed that *circ-Ntrk2* knockdown could prominently inhibit PASMCs proliferation and relieve pulmonary vascular remodelling and right ventricular hypertrophy in vivo and in vitro.

We further predicted the miRNAs targeted by *circ-Ntrk2* through bioinformatics analysis and showed that *circ-Ntrk2* could directly target *miR-296-5p*. Studies have shown that *miR-296-5p* is involved in various diseases. Li et al. [37, 38] reported that *miR-296-5p* regulates gastric cancer cell proliferation. Shi et al. [39] reported that *miR-296-5p* overexpression inhibits hepatocellular carcinoma progression. However, changes in the expression and role of *miR-296-5p* in PAH have not been confirmed. Our study is the first to demonstrate that *miR-296-5p* is downregulated in hypoxic PAH and that its overexpression inhibits PASMC proliferation and mediates the regulatory mechanism of *circ-Ntrk2* in PAH.

The important role of the TGF pathway in PAH has been well established [40–43]. Cai et al. [44] reported that TGF-β1 activation could induce excessive PASMC proliferation and reduce apoptosis through STAT3, thereby driving pulmonary vascular remodelling. Moreover, p38 MAPK is widely recognised as a negative factor in PAH [45, 46], and the presence of TGF-β1/p38 MAPK pathway has been reported in numerous diseases, including PAH [47–49]. Therefore, we further investigated whether the TGF-β1/p38 MAPK pathway is involved in the process by which *circ-Ntrk2* affects PAH. Interestingly, the bioinformatic analysis showed that TGF-β1 is a potential downstream target of *miR-296-5p*. We also demonstrated the targeted regulatory relationship between *miR-296-5p* and TGF-β1 and found that HYP-induced TGF-β1 and p38 MAPK upregulation was inhibited by *circ-Ntrk2* knockdown, while *miR-296-5p* supplementation reversed this change. In conclusion, our study showed that antagonising *circ-Ntrk2* could alleviate PAH by inhibiting PASMC proliferation and reversing pulmonary vascular remodelling through the *miR-296-5p*/TGF-β1/p38 MAPK signal transduction axis.

Our study identified a new circRNA (*circ-Ntrk2*) and further explored its function and mechanism, which is an important addition to circRNA research in the field of PAH. However, because of the complexity of the circRNA regulatory network, only the representative network was identified in this study. Whether another *circ-Ntrk2*-miRNA-mRNA regulatory axis exists under our pathological conditions will be the direction of our future studies.

## Conclusions

In this study, we demonstrated that *circ-Ntrk2* expression is upregulated in hypoxic lung tissues and PASMCs and that its deficiency could improve pulmonary vascular remodelling and relieve PAH via the *miR-296-5p*/TGF-β1/p38 MAPK signal transduction axis, thus establishing potential targets for PAH diagnosis and treatment. Furthermore, our study contributes to the understanding of circRNA in relation to PAH.

## Supplementary Information

**Additional file 1.** Measurement of arterial pressure in mice and detection of drug knockout efficiency.

**Additional file 2.** The primary antibodies used in this study.

**Additional file 3.** The primers used in this study.

**Additional file 4.** The gel or blot images.

## Data Availability

All relevant data and materials are stored at the Key Laboratory of Heart and Lung of Wenzhou Medical University and are available from the first author and corresponding author on reasonable request.

## Abbreviations

CircRNA: Circular RNA
ncRNA: Non-coding RNA
miRNA: MicroRNA
PAH: Pulmonary arterial hypertension
PASMCs: Pulmonary artery smooth muscle cells
PBS: Phosphate-buffered saline
PCNA: Proliferating cell nuclear antigen
TGF-β1: Transforming growth factor-β1
NC: Negative control
NOR: Normoxia
HYP: Hypoxia
siRNA: Small interfering RNA
mut: Mutant
RV: Right ventricle
RVSP: Right ventricular systolic pressure
S: Septum
LV: Left ventricle
H&E: Hematoxylin–eosin
WT: Wall thickness
TT: Total thickness
WA: Pulmonary artery wall area
PAAT: Pulmonary artery acceleration time
PAVTI: Pulmonary artery velocity time integral
gDNA: Genomic DNA
FISH: Fluorescence in situ hybridization
CCK-8: Cell counting kit-8
EdU: 5-Ethynyl-20-deoxyuridine
qRT-PCR: Quantitative real-time polymerase chain reaction
DAPI: 4,6-Diamidino-2-phenylindole
