# Supplementary material for: Circ-Ntrk2 acts as a miR-296-5p sponge to activate the TGF-β1/p38 MAPK pathway and promote pulmonary hypertension and vascular remodelling
Source: Respir Res. 2023 Mar 13;24:78. doi: 10.1186/s12931-023-02385-7 (PMC10012448; doi:10.1186/s12931-023-02385-7)
Supplement: Supplementary file 1 — Additional file 1. Measurement of arterial pressure in mice and detection of drug knockout efficiency. [file 12931_2023_2385_MOESM1_ESM.docx]

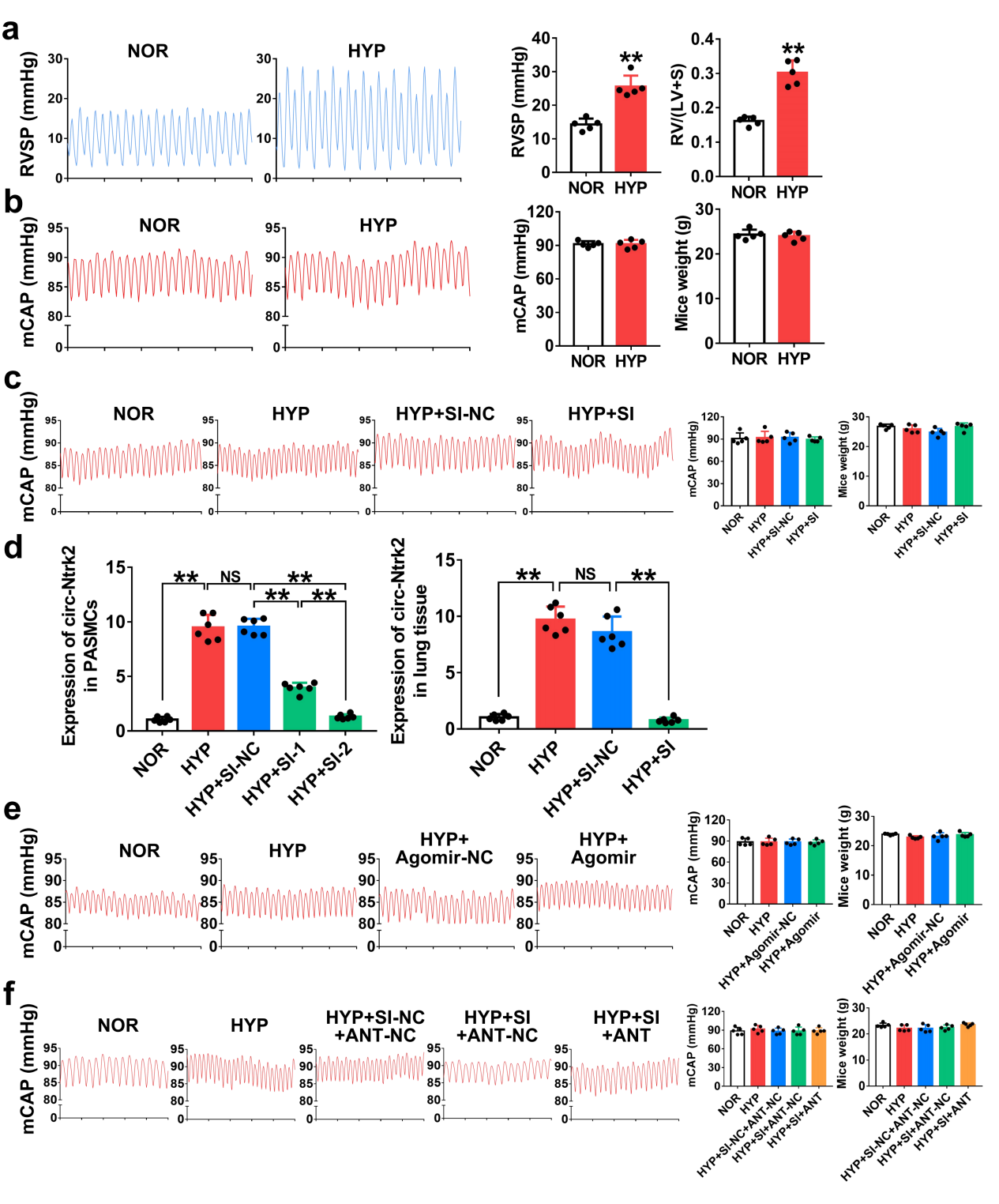


**Supplementary Fig. 1** Measurement of arterial pressure in mice and detection of drug knockout efficiency. **a** Right ventricular systolic pressure (RVSP) and right ventricle (RV)/left ventricle (LV)+septum (S) weight ratio in the NOR and HYP groups (n = 5). **b** Meancervicalarterial pressure (mCAP) and mice weight in the NOR and HYP groups (n = 5). **c** Meancervicalarterial pressure (mCAP) and mice weight in the NOR, HYP, HYP+ SI-NC, and HYP+SI groups (n = 5). **d** qRT-PCR analysis of *circ-Ntrk2* after treatment with siRNA in lung tissue and PASMCs. **e** Meancervicalarterial pressure (mCAP), mice weight in the NOR, HYP, HYP+Agomir-NC, and HYP+Agomir groups (n = 5). **f** Meancervicalarterial pressure (mCAP) and mice weight in the NOR, HYP, HYP+SI-NC+ANT-NC, HYP+SI+ANT-NC, and HYP+SI+ANT groups (n = 5). All values are presented as the mean±SEM. **P<0.05*, ***P<0.01*
