# Supplementary material for: Circ-Ntrk2 acts as a miR-296-5p sponge to activate the TGF-β1/p38 MAPK pathway and promote pulmonary hypertension and vascular remodelling
Source: Respir Res. 2023 Mar 13;24:78. doi: 10.1186/s12931-023-02385-7 (PMC10012448; doi:10.1186/s12931-023-02385-7)
Supplement: Supplementary file 2 — Additional file 2. The primary antibodies used in this study. [file 12931_2023_2385_MOESM2_ESM.docx]

**Supplementary Table 1** The primary antibodies used in this study.

| Antibody | Source | Identifier |
| --- | --- | --- |
| proliferating cell nuclear antigen (PCNA) | Cell Signaling Technology | #13110 |
| cyclin D1 | Affinity Biosciences | #AF0931 |
| transforming growth factor-β (TGF-β1) | Abcam | #ab215715 |
| p38 MAPK | Cell Signaling Technology | #8690 |
| β-tubulin | Cell Signaling Technology | #2128 |
| [Goat Anti-Rabbit IgG H&L (HRP)](https://www.abcam.cn/goat-rabbit-igg-hl-hrp-ab6721.html) | Abcam | #ab6721 |
| Donkey anti-mouse IgG H&L (Alexa Fluor 488) | Abcam | #ab150105 |
| α-smooth muscle actin (α-SMA) | Abcam | #ab5694 |
| Horseradish peroxidase-conjugated goat anti-rabbit IgG | Biosharp | # BL003A |
