# Supplementary material for: Circ-Ntrk2 acts as a miR-296-5p sponge to activate the TGF-β1/p38 MAPK pathway and promote pulmonary hypertension and vascular remodelling
Source: Respir Res. 2023 Mar 13;24:78. doi: 10.1186/s12931-023-02385-7 (PMC10012448; doi:10.1186/s12931-023-02385-7)
Supplement: Supplementary file 3 — Additional file 3. The primers used in this study. [file 12931_2023_2385_MOESM3_ESM.docx]

**Supplementary Table 2** The primers used in this study.

| Target | Primers/siRNAs | Sequence(5’→3’) |
| --- | --- | --- |
| Circ-Ntrk2 | Forward primer | AAGAACGAGTATGGGAAGGATGA |
|  | Reverse primer | TGCAGATGGCAGACCGTAGTC |
| Linear Ntrk2 | Forward primer | CTGGTCTTGGGCTTCTGGAG |
|  | Reverse primer | CGTGATGTTCTCCGGGTCAACG |
| TGF-β1 | Forward primer | GCTAATGGTGGACCGCAAC |
|  | Reverse primer | GCTTCCCGAATGTCTGACGTA |
| β-actin | Forward primer | GATGCTCCCCGGGCTGTA |
|  | Reverse primer | GCCTCGTCACCCACATAGGAGT |
| GAPDH | Forward primer | CATCACTGCCACCCAGAAGACT |
|  | Reverse primer | ATGCCAGTGAGCTTCCCGTTC |
| miR-296-5p | Forward primer | AGTGCAGGGTCCGAGGTATT |
|  | Reverse primer | CCCCCTCAATCCTGTGTCG |
|  | RT primer | GTCGTATCCAGTGCAGGGTCCGAGGTATTCGCACTGGATACGACACAGGAT |
| U6 | Forward primer | AGAGAAGATTAGCATGGCCCCTG |
|  | Reverse primer | ATCCAGTGCAGGGTCCGAGG |
|  | RT primer | GTCGTATCCAGTGCAGGGTCCGAGGTATTCGCACTGGATACGACAAAATA |
| miR-296-5p | Mimic sense | AGGGCCCCCCCUCAAUCCUGU |
|  | Mimic antisense | ACAGGAUUGAGGGGGGGCCCU |
|  | Mimics-NC sense | UUUGUACUACACAAAAGUACUG |
|  | Mimics-NC antisense | CAGUACUUUUGUGUAGUACAAA |
| miR-296-5p | Inhibitor | ACAGGAUUGAGGGGGGGCCCU |
|  | Inhibitor-NC | CAGUACUUUUGUGUAGUACAAA |
| Circ-Ntrk2 | siRNA-NC | GGCTCTAGAAAAGCCTATGC |
|  | siRNA-1 | CTACGGTCTGCCATCTGCA |
|  | siRNA-2 | CGACTACGGTCTGCCATCT |
| Circ-Ntrk2 | probe | CY3-AGATGGCAGACCGTAGTCGACTCCAGG-CY3 |
| miR-296-5p | probe | FAM-ACAGGATTGAGGGGGGGCCCT-FAM |
| Circ-Ntrk2 | pmirGLO-mmu-circ-Ntrk2-WT | GTCTGCCATCTGCACGTCTGGCTGCTCCTAACCTCACCGTGGAGGAAGGAAAGTCTGTGACCCTTTCCTGCAGTGTGGGGGGTGACCCACTCCCCACCTTGTACTGGGACGTTGGGAATTTGGTTTCCAAGCACATGAATGAAACAAGCCACACACAGGGCTCCTTAAGGATAACGAACATTTCATCTGATGACAGTGGAAAGCAAATCTCTTGTGTGGCAGAAAACCTTGTAGGAGAAGATCAAGATTCTGTGAACCTCACTGTGCATTTTGCGCCAACTATCACGTTTCTCGAGTCTCCAACCTCAGATCACCACTGGTGCATTCCATTCACTGTGAGAGGCAACCCCAAGCCTGCGCTTCAGTGGTTCTACAATGGGGCCATACTGAATGAGTCCAAGTACATCTGTACTAAGATCCACGTCACCAATCACACGGAGTACCATGGCTGCCTCCAGCTGGATAACCCCACTCATATGAATAACGGAGACTACACCCTGATGGCCAAGAACGAGTATGGGAAGGATGAGAGACAGATCTCCGCTCACTTCATGGGCCGGCCTGGAGTCGACTACG |
| Circ-Ntrk2 | pmirGLO-mmu-circ-Ntrk2-MUT（69 and 362 position） | GTCTGCCATCTGCACGTCTGGCTGCTCCTAACCTCACCGTGGAGGAAGGAAAGTCTGTGACCCTTTCCTCGTCACTCCCCCCTCAGGGACTCCCCACCTTGTACTGGGACGTTGGGAATTTGGTTTCCAAGCACATGAATGAAACAAGCCACACACAGGGCTCCTTAAGGATAACGAACATTTCATCTGATGACAGTGGAAAGCAAATCTCTTGTGTGGCAGAAAACCTTGTAGGAGAAGATCAAGATTCTGTGAACCTCACTGTGCATTTTGCGCCAACTATCACGTTTCTCGAGTCTCCAACCTCAGATCACCACTGGTGCATTCCATTCACTGTGAGAGGCAACCCCAAGCCTGCGCTTGTCTCCAACTACAATCCCCGGATACTGAATGAGTCCAAGTACATCTGTACTAAGATCCACGTCACCAATCACACGGAGTACCATGGCTGCCTCCAGCTGGATAACCCCACTCATATGAATAACGGAGACTACACCCTGATGGCCAAGAACGAGTATGGGAAGGATGAGAGACAGATCTCCGCTCACTTCATGGGCCGGCCTGGAGTCGACTACG |
| Circ-Ntrk2 | pmirGLO-mmu-circ-Ntrk2-MUT（69 and 553 position） | GTCTGCCATCTGCACGTCTGGCTGCTCCTAACCTCACCGTGGAGGAAGGAAAGTCTGTGACCCTTTCCTCGTCACTCCCCCCTCAGGGACTCCCCACCTTGTACTGGGACGTTGGGAATTTGGTTTCCAAGCACATGAATGAAACAAGCCACACACAGGGCTCCTTAAGGATAACGAACATTTCATCTGATGACAGTGGAAAGCAAATCTCTTGTGTGGCAGAAAACCTTGTAGGAGAAGATCAAGATTCTGTGAACCTCACTGTGCATTTTGCGCCAACTATCACGTTTCTCGAGTCTCCAACCTCAGATCACCACTGGTGCATTCCATTCACTGTGAGAGGCAACCCCAAGCCTGCGCTTCAGTGGTTCTACAATGGGGCCATACTGAATGAGTCCAAGTACATCTGTACTAAGATCCACGTCACCAATCACACGGAGTACCATGGCTGCCTCCAGCTGGATAACCCCACTCATATGAATAACGGAGACTACACCCTGATGGCCAAGAACGAGTATGGGAAGGATGAGAGACAGATCTCCGCTCACTTCATCCCCCCCGGAGGAGTCGACTACG |
| Circ-Ntrk2 | pmirGLO-mmu-circ-Ntrk2-MUT（362 and 553 position） | GTCTGCCATCTGCACGTCTGGCTGCTCCTAACCTCACCGTGGAGGAAGGAAAGTCTGTGACCCTTTCCTGCAGTGTGGGGGGTGACCCACTCCCCACCTTGTACTGGGACGTTGGGAATTTGGTTTCCAAGCACATGAATGAAACAAGCCACACACAGGGCTCCTTAAGGATAACGAACATTTCATCTGATGACAGTGGAAAGCAAATCTCTTGTGTGGCAGAAAACCTTGTAGGAGAAGATCAAGATTCTGTGAACCTCACTGTGCATTTTGCGCCAACTATCACGTTTCTCGAGTCTCCAACCTCAGATCACCACTGGTGCATTCCATTCACTGTGAGAGGCAACCCCAAGCCTGCGCTTGTCTCCAACTACAATCCCCGGATACTGAATGAGTCCAAGTACATCTGTACTAAGATCCACGTCACCAATCACACGGAGTACCATGGCTGCCTCCAGCTGGATAACCCCACTCATATGAATAACGGAGACTACACCCTGATGGCCAAGAACGAGTATGGGAAGGATGAGAGACAGATCTCCGCTCACTTCATCCCCCCCGGAGGAGTCGACTACG |
| Circ-Ntrk2 | pmirGLO-mmu-circ-Ntrk2-MUT（69, 362 and 553 position） | GTCTGCCATCTGCACGTCTGGCTGCTCCTAACCTCACCGTGGAGGAAGGAAAGTCTGTGACCCTTTCCTCGTCACTCCCCCCTCAGGGACTCCCCACCTTGTACTGGGACGTTGGGAATTTGGTTTCCAAGCACATGAATGAAACAAGCCACACACAGGGCTCCTTAAGGATAACGAACATTTCATCTGATGACAGTGGAAAGCAAATCTCTTGTGTGGCAGAAAACCTTGTAGGAGAAGATCAAGATTCTGTGAACCTCACTGTGCATTTTGCGCCAACTATCACGTTTCTCGAGTCTCCAACCTCAGATCACCACTGGTGCATTCCATTCACTGTGAGAGGCAACCCCAAGCCTGCGCTTGTCTCCAACTACAATCCCCGGATACTGAATGAGTCCAAGTACATCTGTACTAAGATCCACGTCACCAATCACACGGAGTACCATGGCTGCCTCCAGCTGGATAACCCCACTCATATGAATAACGGAGACTACACCCTGATGGCCAAGAACGAGTATGGGAAGGATGAGAGACAGATCTCCGCTCACTTCATCCCCCCCGGAGGAGTCGACTACG |
