# Supplementary material for: Circ-Ntrk2 acts as a miR-296-5p sponge to activate the TGF-β1/p38 MAPK pathway and promote pulmonary hypertension and vascular remodelling
Source: Respir Res. 2023 Mar 13;24:78. doi: 10.1186/s12931-023-02385-7 (PMC10012448; doi:10.1186/s12931-023-02385-7)
Supplement: Supplementary file 4 — Additional file 4. The gel or blot images. [file 12931_2023_2385_MOESM4_ESM.pptx]

## Slide 1
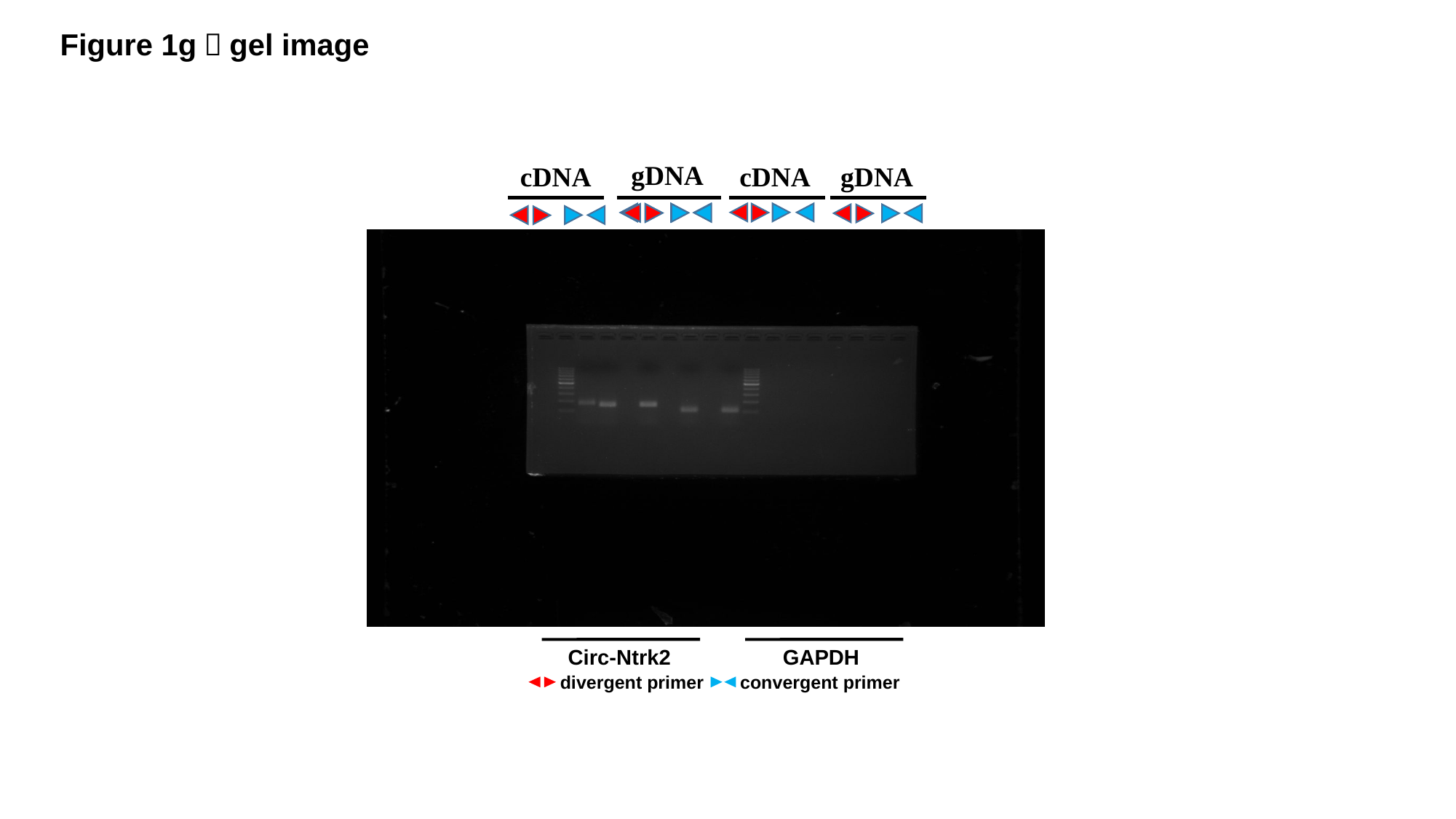

Figure 1g：gel image
gDNA
cDNA
cDNA
gDNA
Circ-Ntrk2
GAPDH
divergent primer
convergent primer

## Slide 2
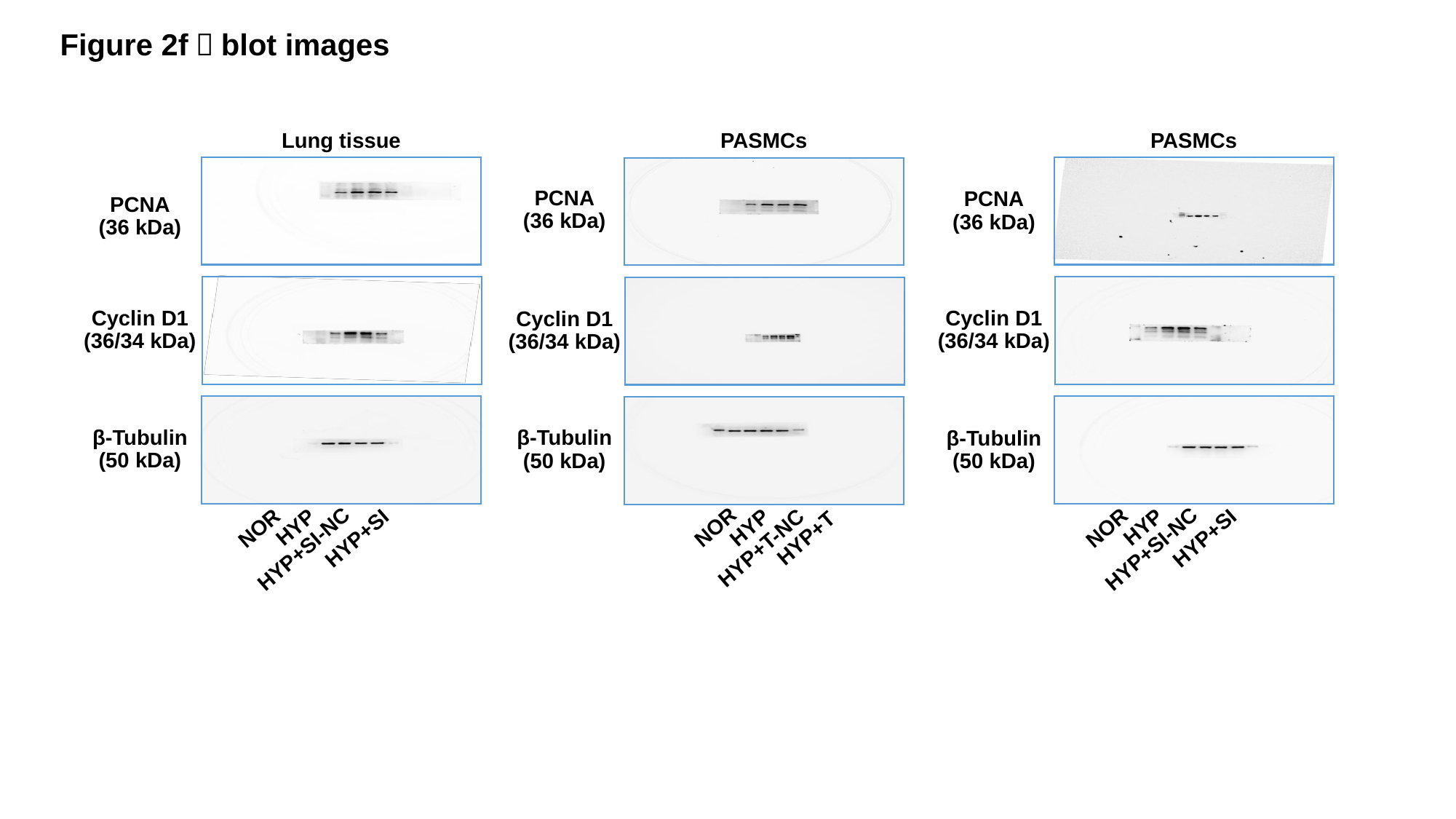

Figure 2f：blot images
Lung tissue
PASMCs
PASMCs
PCNA
(36 kDa)
PCNA
(36 kDa)
PCNA
(36 kDa)
Cyclin D1
(36/34 kDa)
Cyclin D1
(36/34 kDa)
Cyclin D1
(36/34 kDa)
β-Tubulin
(50 kDa)
β-Tubulin
(50 kDa)
β-Tubulin
(50 kDa)
HYP
HYP
NOR
HYP
NOR
NOR
HYP+SI
HYP+SI
HYP+T
HYP+T-NC
HYP+SI-NC
HYP+SI-NC

## Slide 3
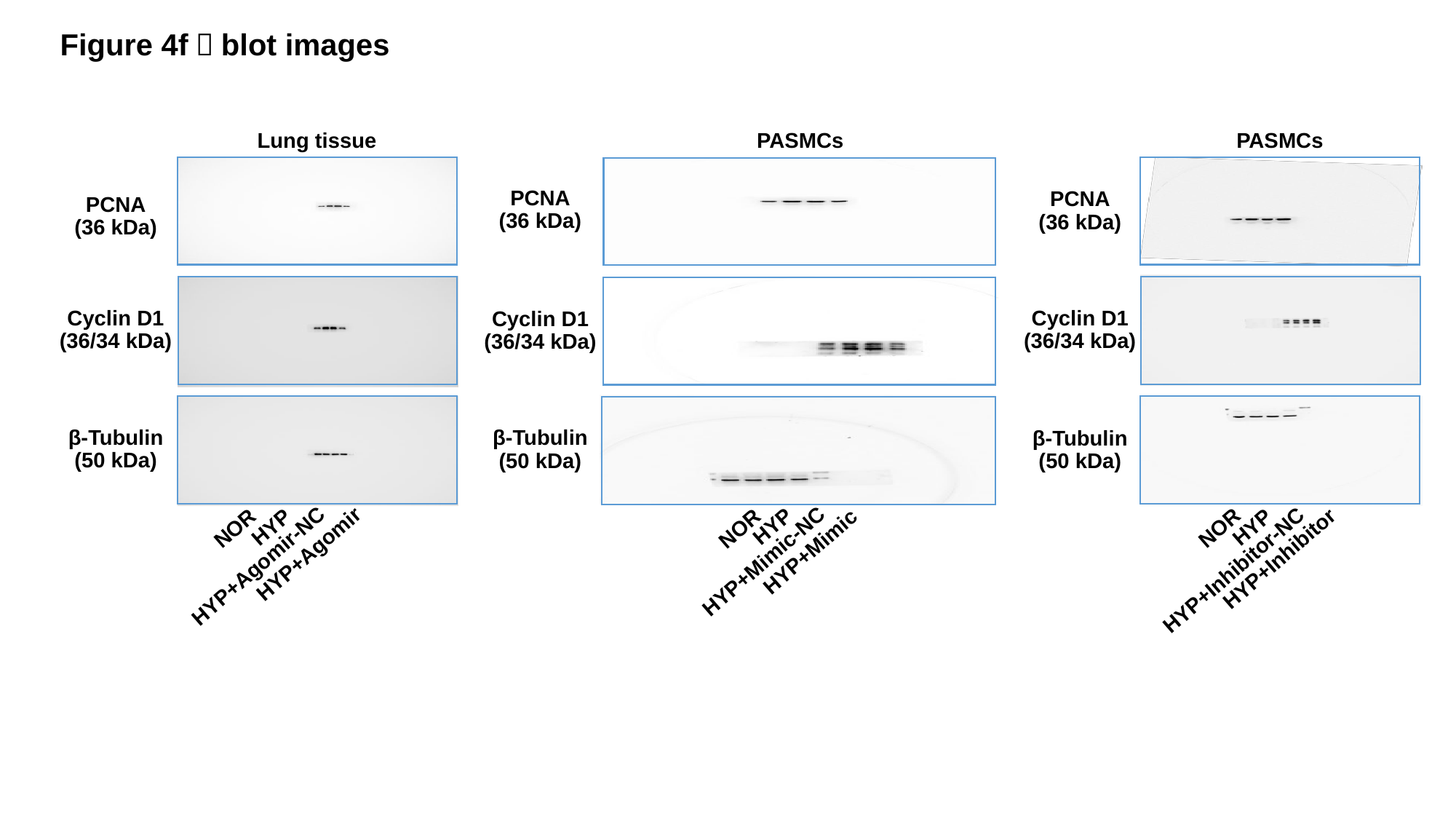

Figure 4f：blot images
Lung tissue
PASMCs
PASMCs
PCNA
(36 kDa)
PCNA
(36 kDa)
PCNA
(36 kDa)
Cyclin D1
(36/34 kDa)
Cyclin D1
(36/34 kDa)
Cyclin D1
(36/34 kDa)
β-Tubulin
(50 kDa)
β-Tubulin
(50 kDa)
β-Tubulin
(50 kDa)
HYP
HYP
HYP
NOR
NOR
NOR
HYP+Mimic
HYP+Agomir
HYP+Inhibitor
HYP+Mimic-NC
HYP+Agomir-NC
HYP+Inhibitor-NC

## Slide 4
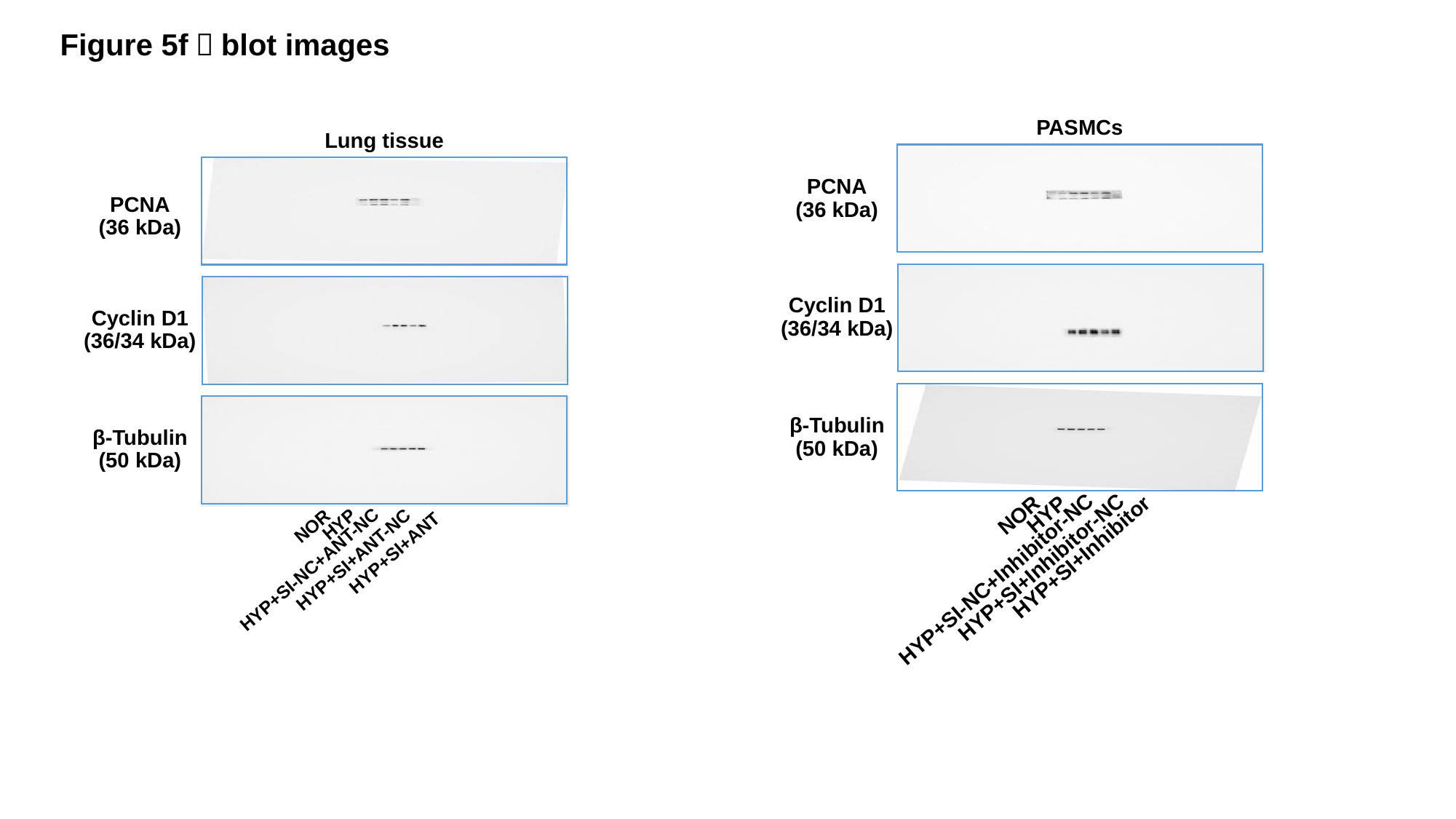

Figure 5f：blot images
PASMCs
Lung tissue
PCNA
(36 kDa)
PCNA
(36 kDa)
Cyclin D1
(36/34 kDa)
Cyclin D1
(36/34 kDa)
β-Tubulin
(50 kDa)
β-Tubulin
(50 kDa)
HYP
NOR
HYP
NOR
HYP+SI+ANT
HYP+SI+Inhibitor
HYP+SI+ANT-NC
HYP+SI+Inhibitor-NC
HYP+SI-NC+ANT-NC
HYP+SI-NC+Inhibitor-NC

## Slide 5
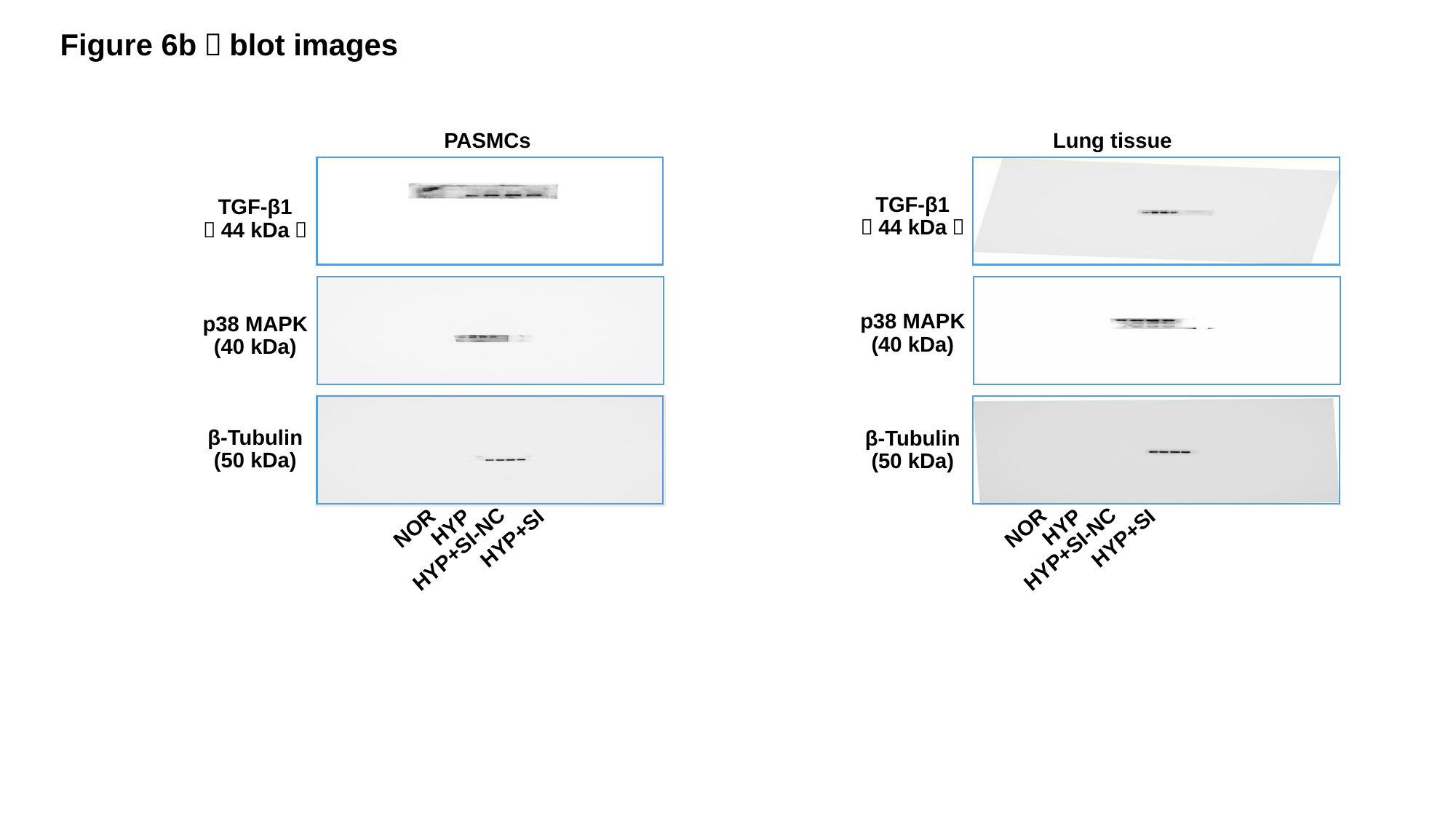

Figure 6b：blot images
PASMCs
Lung tissue
TGF-β1
（44 kDa）
TGF-β1
（44 kDa）
p38 MAPK
(40 kDa)
p38 MAPK
(40 kDa)
β-Tubulin
(50 kDa)
β-Tubulin
(50 kDa)
HYP
HYP
NOR
NOR
HYP+SI
HYP+SI
HYP+SI-NC
HYP+SI-NC

## Slide 6
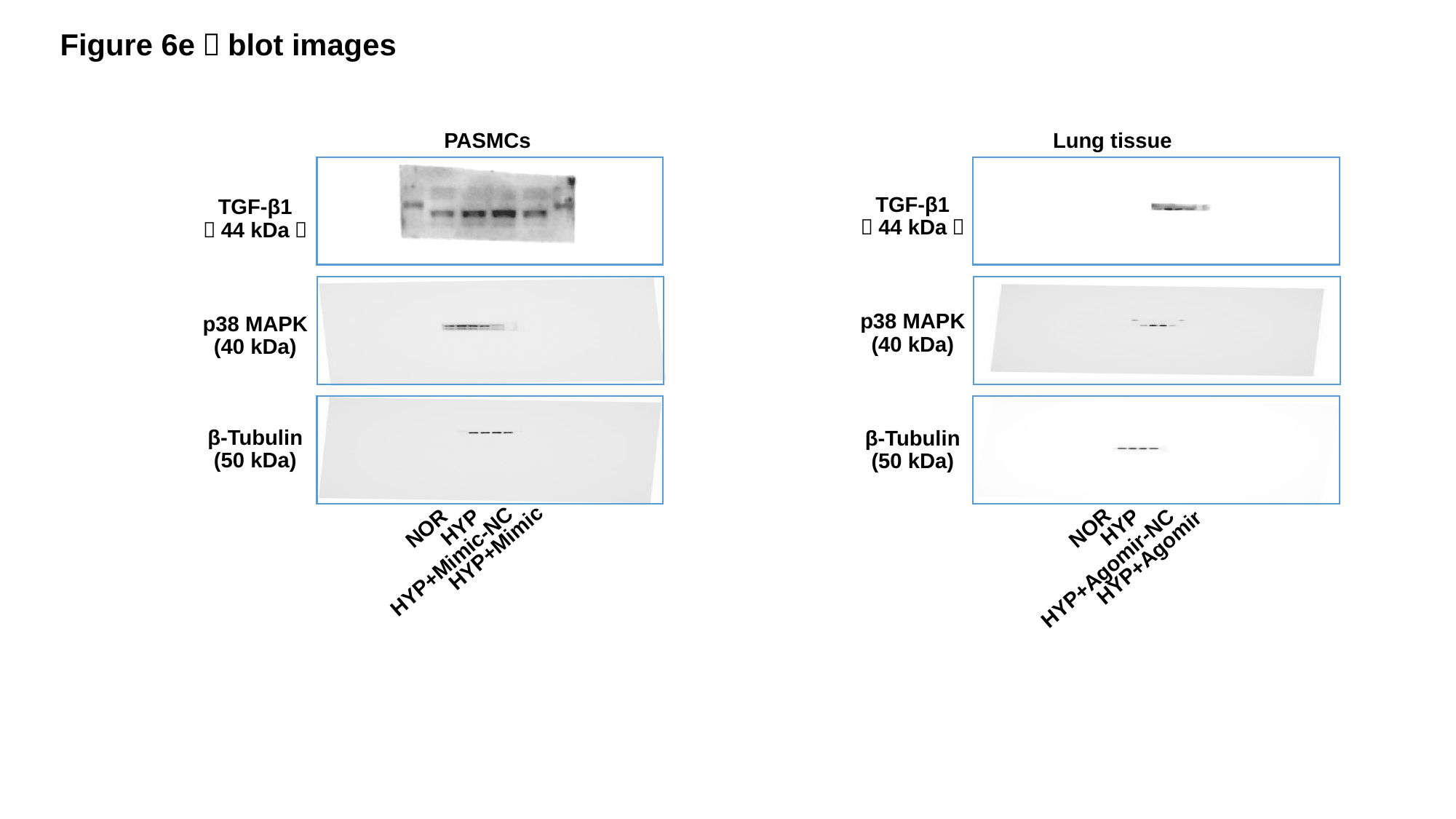

Figure 6e：blot images
PASMCs
Lung tissue
TGF-β1
（44 kDa）
TGF-β1
（44 kDa）
p38 MAPK
(40 kDa)
p38 MAPK
(40 kDa)
β-Tubulin
(50 kDa)
β-Tubulin
(50 kDa)
HYP
HYP
NOR
NOR
HYP+Mimic
HYP+Agomir
HYP+Mimic-NC
HYP+Agomir-NC

## Slide 7
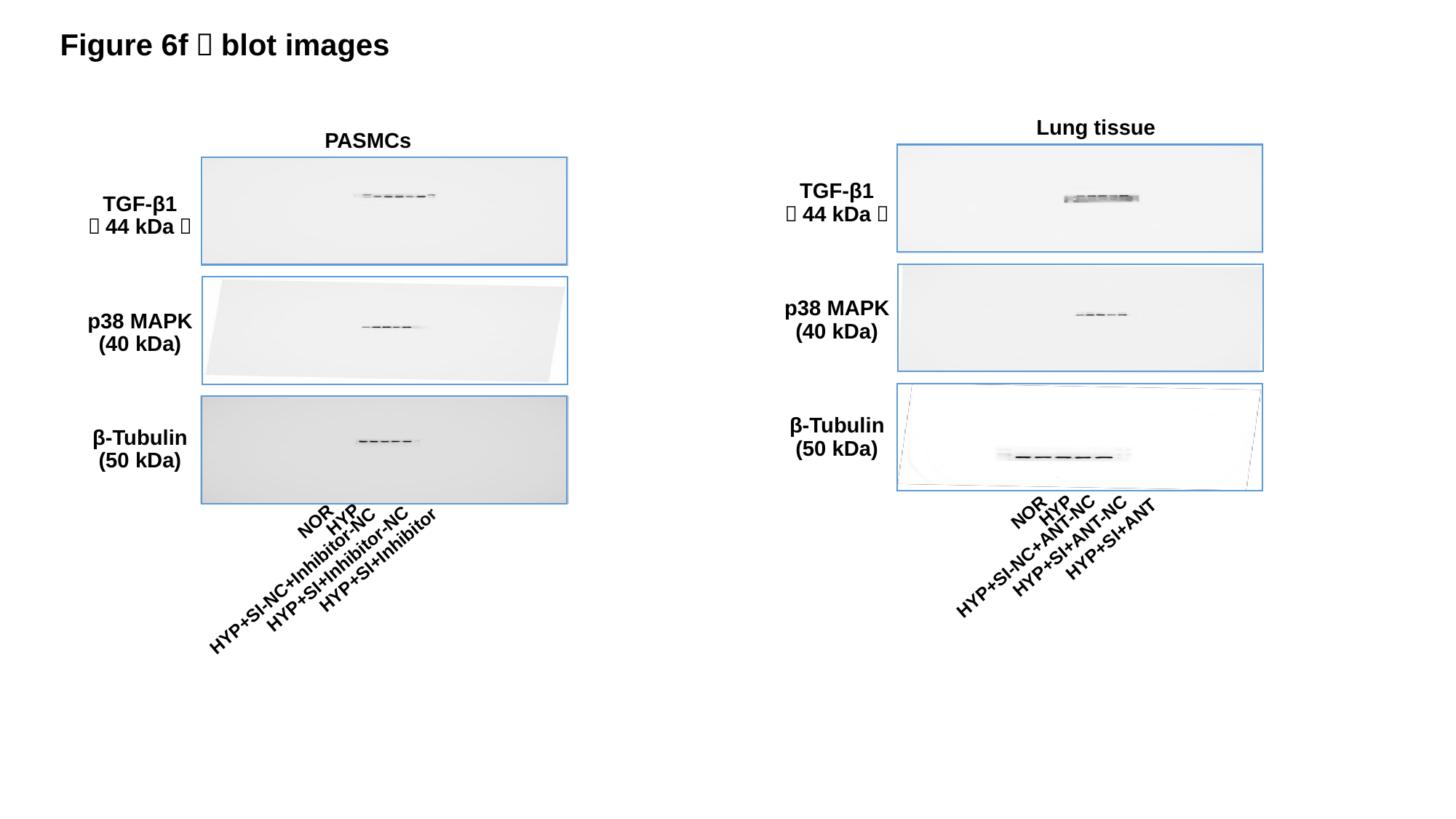

Figure 6f：blot images
Lung tissue
PASMCs
TGF-β1
（44 kDa）
TGF-β1
（44 kDa）
p38 MAPK
(40 kDa)
p38 MAPK
(40 kDa)
β-Tubulin
(50 kDa)
β-Tubulin
(50 kDa)
HYP
NOR
HYP
NOR
HYP+SI+ANT
HYP+SI+ANT-NC
HYP+SI-NC+ANT-NC
HYP+SI+Inhibitor
HYP+SI+Inhibitor-NC
HYP+SI-NC+Inhibitor-NC
